# Gonadotropin-releasing hormone antagonists versus agonists for endometriosis: a systematic review and meta-analysis of randomized controlled trials

**DOI:** 10.3389/fendo.2026.1979544

**Published:** 2026-10-14

**Authors:** Tianmeng Liu, Yuhong Cai, Mingyang Li, Suqing Cai, Leilei Wang, Dongmei Tang, Yiling Wu, Nan Zhu

**Affiliations:** 1 Department of Obstetrics and Gynecology, Xiamen Susong Hospital, Xiamen, Fujian, China; 2 Women and Children’s Hospital Affiliated to Xiamen University, Xiamen Maternal and Child Health Hospital, Xiamen, Fujian, China; 3 Graduate School of Dalian Medical University, Dalian, China

**Keywords:** bone mineral density, endometriosis, gonadotropin-releasing hormone agonists, gonadotropin-releasing hormone antagonists, meta-analysis, pelvic pain

## Abstract

**Background:**

Endometriosis is a chronic estrogen-dependent inflammatory disease requiring individualized long-term hormonal management. Gonadotropin-releasing hormone agonists (GnRH-a) effectively suppress ovarian function but may induce hypoestrogenic adverse effects, whereas oral gonadotropin-releasing hormone antagonists (GnRH-ant) offer rapid, reversible suppression with reduced hormonal fluctuation. However, their comparative efficacy and safety remain unclear.

**Methods:**

We performed a systematic review and meta-analysis of randomized controlled trials comparing GnRH-ant with leuprolide-based GnRH-a for the treatment of endometriosis, assessing symptom relief, pelvic signs, bone mineral density (BMD), quality of life, and hormonal outcomes.

**Results:**

Five randomized controlled trials were included. Compared with GnRH-ant, GnRH-a yielded greater improvements in dysmenorrhea (standardized mean difference (SMD) = 0.69, 95% CI: 0.48–0.91), nonmenstrual pelvic pain (NMPP) (SMD = 0.33, 95% CI: 0.16–0.50), overall pelvic pain (OPP) (SMD = 0.25, 95% CI: 0.13–0.38), and dyspareunia (SMD = 0.14, 95% CI: 0.02–0.26). GnRH-a was also associated with better outcomes in pelvic tenderness, Douglas pouch induration scores, peak pain severity, analgesic requirements, and menstrual blood loss. Subgroup analysis suggested a more pronounced analgesic benefit of GnRH-a after 12 weeks of treatment. In contrast, GnRH-ant resulted in significantly less BMD reduction (SMD = 0.64, 95% CI: 0.39–0.89), a stronger bone-sparing effect at 24 weeks. No significant differences were observed in most quality-of-life domains.

**Conclusions:**

Leuprolide-based GnRH-a provides superior symptom control, whereas GnRH-ant offers less decline in BMD and reversible ovarian suppression, but differences among individual antagonists should be considered. Treatment selection should be individualized based on symptom severity, bone health, and fertility goals. Long-term head-to-head trials are warranted.

**Systematic review registration:**

https://www.crd.york.ac.uk/PROSPERO/view/CRD420261289010, identifier CRD420261289010.

## 1 Introduction

Endometriosis is a chronic, estrogen-dependent disease characterized by the presence of endometrium-like tissue outside the uterine cavity and affects approximately 10% of women of reproductive age (1). Once established, endometriotic lesions may promote persistent inflammation, fibrosis, and neurovascular remodeling, leading to symptoms such as dysmenorrhea, nonmenstrual pelvic pain (NMPP), dyspareunia, and dyschezia. Some patients may also experience infertility, bowel dysfunction, fatigue, weight loss, allergic manifestations, systemic inflammation, and impaired psychosocial functioning (2–4). In recent years, accumulating molecular and clinical evidence has suggested that endometriosis should not be regarded solely as a localized pelvic disorder. Instead, it is increasingly recognized as a disease with systemic features closely associated with immune dysregulation and chronic inflammation, often accompanied by a range of comorbidities (5). A substantial delay often occurs between symptom onset and definitive diagnosis, and the disease may follow a prolonged and recurrent course. The symptoms can persist or recur over time. It is worth noting that the severity of the patient’s pain is not always consistent with the scope of the lesion, which also means that treatment cannot be judged solely based on the size of the lesion or the stage. In clinical practice, it is more necessary to formulate personalized, multimodal and interdisciplinary treatment plans by combining the patient’s symptom characteristics, fertility needs et al (6). Accordingly, the goals of treatment should extend beyond the removal of visible lesions to include symptom control, preservation of quality of life and reproductive potential, and minimization of adverse effects related to long-term therapy.

With continued advances in diagnostic imaging, particularly the increasingly established use of transvaginal ultrasonography and magnetic resonance imaging in clinical practice, the diagnostic approach to endometriosis should now be based on a combination of patient history, clinical examination, and imaging findings, rather than surgery (7). For patients who are not planning pregnancy in the near term, hormonal therapy remains a major strategy for controlling endometriosis-associated pain. Combined hormonal contraceptives and progestogens are generally used as first-line treatments; however, some patients may have an inadequate response, poor tolerance, or contraindications to these therapies. In such cases, further suppression of the hypothalamic–pituitary–ovarian axis may be considered to reduce estrogen production (2). Gonadotropin-releasing hormone agonists (GnRH-a) such as leuprolide initially stimulate pituitary gonadotrophs, resulting in a transient increase in luteinizing hormone (LH), follicle-stimulating hormone (FSH), and sex steroids. With continued administration, pituitary gonadotrophs gradually become functionally desensitized, accompanied by downregulation of GnRH receptors (8). This ultimately suppresses gonadotropin secretion and gonadal steroidogenesis, producing sustained and profound gonadal suppression. GnRH-a are effective in reducing endometriosis-associated pain, but the resulting marked hypoestrogenic state may also lead to hot flushes, vaginal dryness, irregular vaginal bleeding, decreased libido, weight loss, and bone loss. These adverse effects can limit the duration of continuous therapy. Given the need to balance efficacy and safety, the ESHRE guideline recommends GnRH-a as a second-line treatment and advises consideration of add-back hormone therapy to improve tolerability and reduce bone loss and other skeletal safety concerns (2, 9, 10).

In recent years, oral non-peptide gonadotropin-releasing hormone antagonists (GnRH-ant) have increasingly entered clinical practice. GnRH-ant directly and competitively bind to pituitary GnRH receptors without requiring initial receptor stimulation or subsequent receptor desensitization. Suppression of gonadotropin and sex steroid secretion can occur within hours after administration (9), resulting in a relatively rapid onset of action and generally avoiding the initial hormonal flare observed with GnRH-a. The degree of suppression of LH, FSH, and estradiol by GnRH-ant is not uniform and varies according to the specific agent and dose used. After treatment discontinuation, hypothalamic–pituitary–gonadal axis function usually recovers within a relatively short period, indicating that this suppression is readily reversible. Randomized trials of agents such as elagolix and relugolix have shown that GnRH-ant can improve dysmenorrhea and NMPP (11–13). In addition, the degree of estrogen suppression achieved with some GnRH-ant can be modulated by dose. This feature may help preserve analgesic efficacy while reducing bone loss associated with profound hypoestrogenism. However, insufficient suppression of estradiol may also compromise the control of certain pain symptoms or menstrual-related outcomes. Therefore, the clinical use of GnRH-ant still requires a careful balance between treatment efficacy and long-term safety.

Previous studies have predominantly focused on the efficacy of individual GnRH-ant compared with placebo, whereas clinical trials using GnRH-a as active comparators remain relatively limited. Available head-to-head studies have mainly evaluated GnRH-ant such as elagolix, ASP1707, and relugolix, with leuprolide serving as comparator. However, substantial differences across trials in drug dosage, treatment duration, pain assessment instruments, and outcome judgment criteria increase the difficulty of comparing the results of different trials. Some phase II trials suggested that leuprolide may provide greater control of dysmenorrhea or NMPP, whereas GnRH-ant appear to be associated with less bone mineral density (BMD) loss. In contrast, phase III active-controlled trials have shown that relugolix 40 mg once daily is non-inferior to leuprolide in relieving endometriosis-associated pelvic pain (11–14). These findings suggest that the comparative clinical effects of the two drug classes may depend on the specific GnRH-ant, dose, treatment duration, and degree of estradiol suppression. Consequently, the overall benefit–risk balance of GnRH-ant versus GnRH-a cannot be reliably determined from any single trial. Existing systematic reviews have mainly examined GnRH-ant against placebo or indirectly compared different GnRH-ant. To date, evidence from randomized controlled trials directly comparing GnRH-ant with GnRH-a has not been adequately and systematically synthesized.

Accordingly, we systematically searched for and included randomized controlled trials that directly compared GnRH-ant with GnRH-a for the treatment of endometriosis, and conducted a systematic review and meta-analysis of the available evidence. Outcomes of interest included patient-reported measures such as dysmenorrhea, NMPP, OPP, and dyspareunia, as well as pelvic tenderness, induration of the Douglas cavity, analgesic use, menstrual bleeding, BMD and disease-specific quality of life. Subgroup analysis were further performed according to treatment duration and the specific GnRH-ant used. This study aimed to provide a comprehensive comparison of GnRH-ant and GnRH-a with respect to symptom control and hypoestrogenism-related safety, while exploring whether treatment duration and individual GnRH-ant agents modified their relative effects. By clarifying these differences, our findings may provide more targeted evidence to support individualized treatment selection for patients with different pain profiles, skeletal risks, and fertility needs.

## 2 Method

This systematic review and meta-analysis was performed and reported following the Preferred Reporting Items for Systematic Reviews and Meta-Analyses (PRISMA) guidelines (15), with methodological recommendations from the Cochrane Handbook for Systematic Reviews of Interventions also followed during study design and statistical analysis. The review protocol evaluated the therapeutic efficacy of GnRH-ant and GnRH-a in patients with endometriosis. The protocol was registered in the International Prospective Register of Systematic Reviews (PROSPERO) on 20 January, 2026 (registration number: CRD420261289010).

### 2.1 Search strategy

To retrieve direct comparative evidence regarding GnRH-a versus GnRH-ant in the management of endometriosis, we performed systematic searches across PubMed, Embase, the Cochrane Library, and Web of Science. The retrieval scope of all the above databases is from the establishment of the databases to December 22, 2025. Our search strategy integrated both controlled vocabulary (MeSH and Emtree) and free-text keywords. Core search terms included, “Gonadotropin-Releasing Hormone/agonists,” “GnRH agonist*,” “Leuprolide,” “Goserelin,” “Triptorelin Pamoate,” “Gonadotropin-Releasing Hormone/antagonists and inhibitors,” “GnRH antagonist*,” “Elagolix,” “Relugolix,” and “Linzagolix,” together with disease-related terms such as “Endometriosis,” “Endometrioma,” and “pelvic pain,” as well as study design terms including “Randomized Controlled Trial” and “Controlled Clinical Trial.” We additionally searched ClinicalTrials.gov to capture unpublished and ongoing trials. Manual screening of reference lists from all eligible studies was conducted to identify potential missed publications. The full search algorithm is detailed in the **Supplementary Material**.

### 2.2 Eligibility criteria

Study participants included premenopausal women aged 18 years or older with regular menstrual cycles and a confirmed diagnosis of endometriosis within five years prior to trial enrollment. All eligible patients presented with typical endometriosis-related symptoms, mainly dysmenorrhea and chronic pelvic pain.

### 2.3 Exclusion criteria

Studies were excluded based on the following criteria: non-randomized controlled study design; participants who received endocrine-interfering gynecological therapies or endometriosis-related surgery within four weeks prior to trial commencement; abdominal pain caused by alternative etiologies, such as pelvic inflammatory disease or irritable bowel syndrome; a history of hysterectomy or bilateral oophorectomy; uterine fibroids requiring further treatment; uncontrolled systemic disorders involving thyroid, cardiac, hepatic, renal, or hematological dysfunction.

### 2.4 Data extraction

Two trained reviewers independently performed data extraction using a predefined standard form. All extracted data were cross-checked to ensure accuracy and completeness. Missing or incompletely reported data were recorded, and we contacted the corresponding authors to obtain supplementary information when necessary. Disagreements during extraction and assessment were resolved by discussion, with unresolved conflicts adjudicated by a senior third reviewer. We extracted basic study information, participant baseline characteristics, and detailed treatment regimens for both groups, including the specific GnRH-a and GnRH-ant agents, administration routes, and dosages. All outcomes were divided into primary and secondary indicators. The primary outcomes included the relief of dysmenorrhea, NMPP, OPP, dyspareunia, and pelvic tenderness after treatment. Secondary outcomes included changes in induration of Douglas cavity score, peak endometriosis pain score, percentage of days requiring analgesic medication, menstrual blood loss, BMD and Endometriosis Health Profile-5 (EHP-5) scores. These outcomes were assessed using the visual analog scale (VAS), numerical rating scale (NRS), modified Biberoglu and Behrman (M-B&B) score, and the Biberoglu and Behrman (B&B) score, or other corresponding instruments used in the original studies. Detailed data extraction results can be found in **Supplementary Tables 1**-**15**.

### 2.5 Risk of bias assessment

Two independent reviewers evaluated the risk of bias using the Cochrane Collaboration’s Risk of Bias tool (version 1.0). All assessments were cross-checked afterward, and discrepancies were resolved via discussion or adjudicated by a third reviewer when consensus could not be achieved. Seven bias domains were evaluated: random sequence generation, allocation concealment, blinding of participants and personnel, blinding of outcome assessment, incomplete outcome data, selective reporting, and other bias. Each domain was rated as a “low risk,” “high risk,” or “unclear risk” of bias. Detailed assessment results are shown in **Figure 1**.

**Figure 1:**
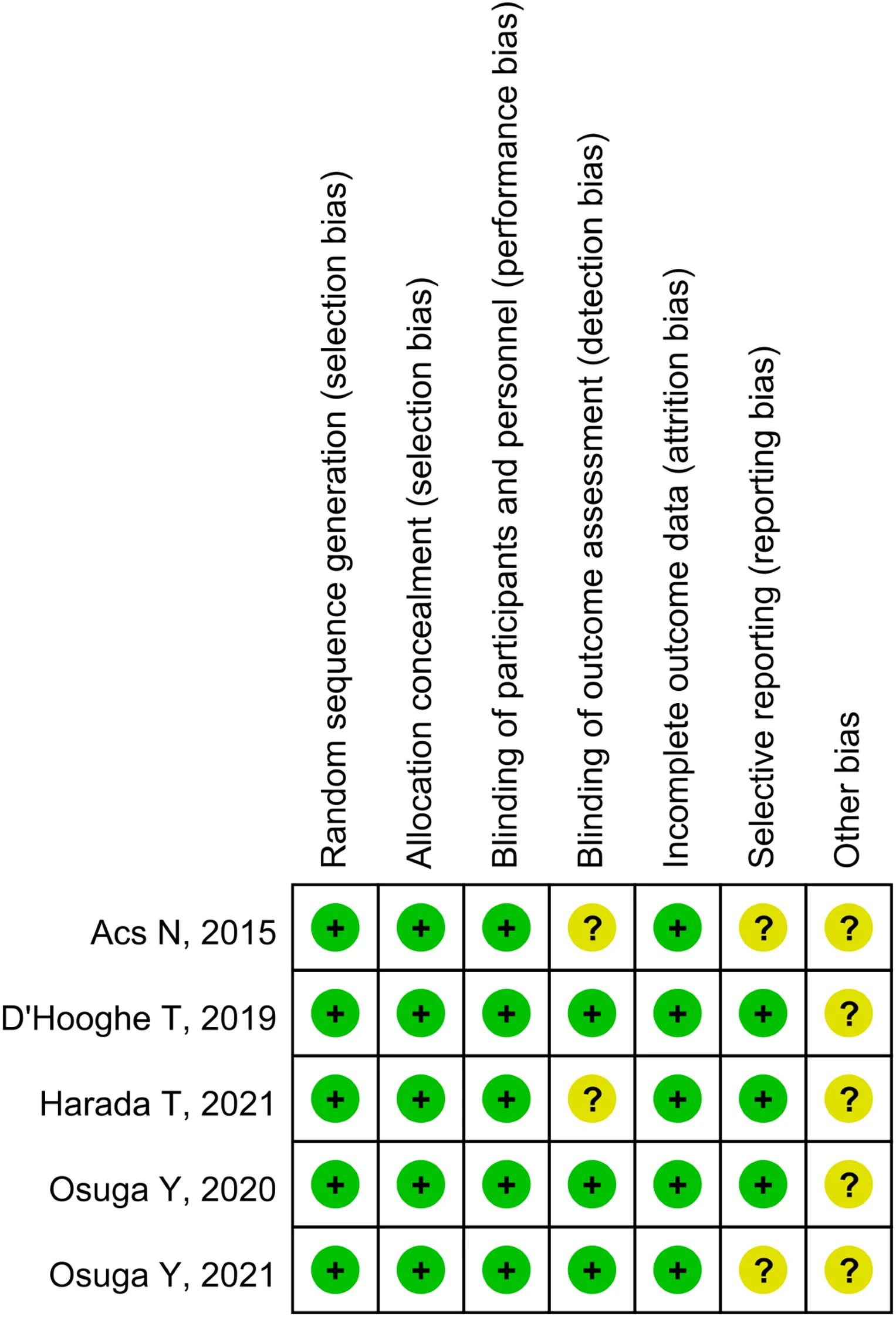
Risk-of-bias assessment of the included studies.

### 2.6 Statistical analysis

All statistical analysis were conducted using RStudio 4.5.2. Given that all included GnRH-ant share a common mechanism of competitive GnRH receptor blockade and are intended to achieve estrogen suppression in endometriosis management, these agents were analyzed as a single therapeutic class. For continuous outcomes including pain scores and BMD, effect indices were selected based on original study measurement approaches. Standardized mean differences (SMDs) with 95% CIs were adopted for outcomes assessed via inconsistent scales or units across trials, while mean differences (MDs) with 95% CIs were used for uniformly measured indicators. As all continuous outcomes were analyzed as changes from baseline, for pain-related outcomes, more negative change scores indicate greater symptom improvement. In contrast, for BMD outcomes, more negative change scores indicate greater bone loss. Between-study heterogeneity was evaluated using Cochran’s Q test and the I² statistic. An I² value <25% was considered to indicate low heterogeneity, 25%–50% moderate heterogeneity, and >50% substantial heterogeneity. Both I² values and the corresponding P values reported. Potential heterogeneity sources were explored regarding patient characteristics, treatment regimens, duration and outcome assessment methods when significant inconsistency existed. The choice of statistical model was predefined based on both anticipated clinical heterogeneity and observed statistical heterogeneity. Fixed-effect models were applied for outcomes with negligible heterogeneity, whereas random-effects models were used when substantial heterogeneity was identified. A two-tailed P value <0.05 was considered statistically significant.

## 3 Results

### 3.1 Study selection

The study screening procedure is illustrated in **Figure 2**. The initial search identified 312 records, and 233 articles remained after duplicate elimination. Following title and abstract screening, 200 records were excluded. Full-text evaluation was performed for the remaining 31 articles, among them, no full text (n = 4), and 27 were further excluded for the following reasons: wrong population (n = 2), wrong study design (n = 6), wrong comparison (n = 4), wrong intervention (n = 2), no data of interest (n = 8). Finally, five studies were included in the subsequent analyses. Basic characteristics of the included studies are summarized in **Table 1**.

**Figure 2:**
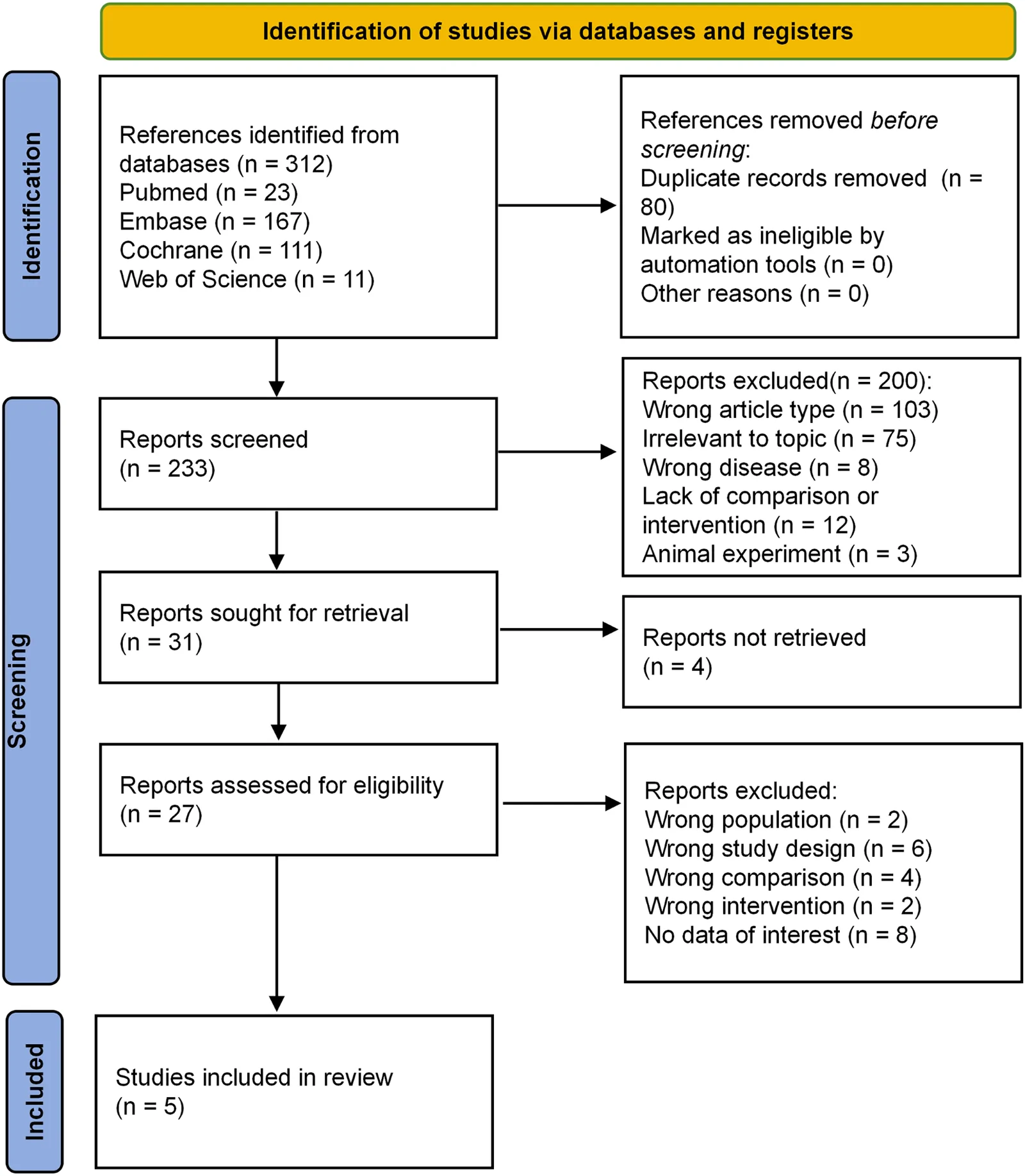
Flow diagram of the study selection process.

**Table 1:** Characteristics of the included studies.

Author	Year	Country	Study design	Intervention drugs	Results
Acs N (11)	2015	27 centers in Central Eastern Europe (Bulgaria, Hungary, Poland, Romania, Russia and Ukraine).	Phase 2, multicenter, randomized, double-blind. Clinical Trial Registration Number: NCT00797225.	Experimental Groups: Elagolix 150 or 250 mg once daily oral tablets. Control Groups: 3.75 mg LA intramuscularly (i.m.) monthly for 12 weeks. LA patients were re-randomized to elagolix, and elagolix patients continued treatment for another 12 weeks.	The changes from baseline in monthly mean values of the NRS for overall endometriosis-associated pelvic pain and modified Biberoglu-Behrman scale for dysmenorrhea, NMPP, dyspareunia; the percentage of days of analgesic use; uterine bleeding and menstrual cycle assessment; BMD of the spine and femur (total hip); treatment-emergent adverse events; EHP-5 score; estradiol levels.
D’Hooghe T (12)	2019	Europe and Japan.	Phase 2, multicenter, randomized, double-blind, parallel-group, placebo-controlled study. Clinical Trial Registration Number: NCT01767090.	Experimental Groups: All patients randomized to ASP1707 took 3 tablets once daily in the morning for 12 weeks (ASP1707–15 mg group: 3 × 5 mg tablets; ASP1707–10 mg group: 2 × 5 mg tablets and 1 placebo tablet; ASP1707–5 mg group: 1 × 5 mg tablet and 2 placebo tablets; ASP1707–3 mg group: 3 × 1 mg tablets). Control Groups: leuprorelin acetate (subcutaneous injection; 3.75 mg/month). During Part 2, subjects received daily oral ASP1707 (3, 5, 10 or 15 mg) or leuprorelin acetate (subcutaneous injection; 3.75 mg/month) for 12 weeks.	The change from baseline to EoT Part 1 and 2 in the mean NRS for OPP, dysmenorrhea, and NMPP; the change from baseline to EoT (for Parts 1 and 2) in the mean NRS relating to interference of pain with daily activities; mean NRS for dyspareunia; mean scores of the M-B&B symptom (i.e. dysmenorrhea, NMPP, and dyspareunia) and sign (i.e. pelvic tenderness and induration) domains;use of rescue medication; mean pain interference score of the Brief Pain Inventory; EHP-5 score; Female Sexual Function Index (FSFI) score; Beck Depression Inventory-II (BDI-II) score; the European quality of life 5-dimension 5-level scale (EQ-5D-5L) score; total plasma ASP1707 concentration; E2, bone alkaline phosphatase (bALP) and collagen type 1 cross-linked N-telopeptide, FSH and LH, and progesterone; adverse events (AEs), safety laboratory tests, vital signs, weight, 12-lead electrocardiograms, physical and gynecological examination, bleeding patterns, hot flashes, and return of menstruation; BMD and trabecular bone score.
Osuga Y (13)	2020	Japan	Phase 2, multicenter, randomized, double-blind, parallel-group, placebo-controlled study. Clinical trial registration number: NCT01458301.	Experimental Groups: Relugolix 10 mg, 20 mg, or 40 mg once daily oral tablets, with leuprorelin-placebo subcutaneous injections; Control Groups: Placebo oral tablets with leuprorelin-placebo subcutaneous injections; or Leuprorelin 3.75 mg subcutaneous injections every 4 weeks with relugolix-placebo oral tablets.	The change from baseline in mean VAS score for pelvic pain during the 28 days before the end of the treatment period; the change from baseline in VAS scores for pelvic pain and dyspareunia during the treatment period; TEAEs, BMD assessed by dualenergy x-ray absorptiometry, vital signs, changes in body weight, 12-lead electrocardiogram, and clinical laboratory test results; M-B&B score for pelvic pain during dysmenorrhea and deep dyspareunia; B&B score for dysmenorrhea, dyspareunia, pelvic pain, pelvic tenderness, and induration; analgesic use during the treatment period; the Endometriosis Health Profile-30 (EHP-30); serum concentrations of estradiol, LH, FSH, and progesterone;
Osuga Y (16)	2021	Japan	Phase 2, multicenter, long term extension study (Open-label extension of a preceding RCT). RCT registration number: NCT01452685.	Experimental Groups: Relugolix 10 mg, 20 mg, or 40 mg daily oral tablets (continued from preceding study); Control Groups: Placebo oral tablets (continued from preceding study); or Leuprorelin 3.75 mg subcutaneous injections every 4 weeks (continued from preceding study).	BMD, TEAEs, vital signs, weight, ECG, and clinical laboratory tests; VAS scores for pelvic pain, dysmenorrhea, and dyspareunia; M-B&B and B&B scales for endometriosis-associated pain symptoms; use of analgesics; decrease in menstrual blood loss; achievement of amenorrheic state; EHP-30; blood concentration of E2, P, LH, and FSH.
Harada T (14)	2021	Japan	Phase 3, multicenter, randomized, double-blind, double-dummy, active-controlled Clinical trial registration number: NCT03931915.	Experimental Groups: 40 mg of REL was orally administered once a day for 24 weeks; Control Groups: 3.75 or 1.88 mg of LEU was subcutaneously injected every 4 weeks for 24 weeks.	The change in the maximum VAS score from baseline to the EOT for endometriosis-associated pelvic pain. the change from baseline to the EOT in the mean VAS score, menstrual pain, NMPP, and dyspareunia; the B&B score, EHP-30 score, and Work Productivity and Activity Impairment Questionnaire: General Health score; uterine mobility restriction, degree of pelvic tenderness, induration of the Douglas cavity, thickness of the endome- trium, size of the ovarian endometrioma, the menstrual period; the serum hormone levels of estradiol (E2), progesterone, LH, and FSH; TEAEs, vital signs, changes in body weight, 12-lead electrocardiogram, clinical laboratory test results, BMD, bone turnover markers (cross- linked N-telopeptide of type 1 collagen and bone alkaline phosphatase), menstruation recovery.

### 3.2 Characteristics of the included RCTs

**Table 1** summarizes the baseline features of eligible trials comparing GnRH-a with GnRH-ant for endometriosis treatment. These studies were conducted across multiple regions, covering Europe, the Middle East, and Japan, and most adopted multicenter, randomized, double-blind controlled designs. All control groups received leuprolide, while intervention regimens varied, mainly involving elagolix, ASP1707, and relugolix, with several trials investigating different drug doses. Primary outcome assessments focused on endometriosis-associated pain, including dysmenorrhea, NMPP, OPP, dyspareunia and pelvic tenderness, measured via NRS, VAS, the M-B&B score, and the B&B score across different trials. Secondary outcomes covered the percentage of days on which analgesic medications were used, menstrual characteristics, BMD, changes in EHP-5 scores. The exclusion criteria were generally similar across the included studies, as detailed in **Supplementary Table 16**. All trials adopted comparable exclusion standards to control confounding bias. Patients with interfering comorbidities, recent related medical treatment, or a history of pelvic endometriosis surgery were generally excluded to ensure accurate efficacy evaluation.

### 3.3 Risk-of-bias assessment

The risk of bias in included RCTs was assessed using the Cochrane-recommended risk-of-bias tool across seven domains, as shown in **Figure 1**. All trials exhibited low selection bias, with adequate random sequence generation and allocation concealment. Participant and personnel blinding was adequately implemented in all of studies. Outcome assessor blinding was clearly reported in many studies, while a number of trials did not clearly report whether outcome assessors were blinded. Consequently, the potential influence of detection bias on outcome measurements could not be completely excluded. Attrition bias (incomplete outcome data) was low risk in most studies, suggesting appropriate handling of missing data. For selective reporting, some studies were unclear due to absent protocols or insufficient outcome reporting, requiring additional information to confirm. Finally, the risk of other bias was judged to be unclear in all included studies, mainly because the original reports provided insufficient information regarding potential conflicts of interest, baseline imbalances, or other factors that might have affected the study findings. Thus, other potential sources of bias could not be definitively identified or excluded. For domains judged as having an unclear risk of bias, the reasons for these judgments were recorded and are provided in **Supplementary Table 17**.

### 3.4 Changes in dysmenorrhea

#### 3.4.1 Subgroup analysis by treatment duration

Subgroup analysis based on treatment duration were performed to evaluate changes in dysmenorrhea severity between GnRH-ant and GnRH-a groups (**Figure 3**). At 12 weeks of treatment, pooled analysis using a random-effects model yielded an SMD of 0.69 (95% CI: 0.45 to 0.94). Compared with the GnRH-a group, the GnRH-ant group presented a smaller reduction in dysmenorrhea scores, indicating superior pain relief in the control group, with substantial inter-study heterogeneity (I² = 81.3%, τ² = 0.1102, P < 0.0001). Similarly, the 24-week subgroup produced a pooled SMD of 0.69 (95% CI: 0.14 to 1.24), also suggested less favorable pain improvement in the GnRH-ant group, accompanied by high heterogeneity (I² = 89.8%, τ² = 0.2133, P < 0.0001), favoring the GnRH-a group for dysmenorrhea relief. Subgroup interaction comparison revealed no statistical difference in effect sizes between 12-week and 24-week regimens (χ² = 0.00, df = 1, P = 0.9876). Leave-one-out sensitivity analysis confirmed the robustness of the pooled estimate, with no individual study substantially influencing the overall effect (**Supplementary Figure 1**). However, since antagonist type, dosage and study design may bring confounding effects, caution should be exercised in interpreting the results of the exploratory subgroup analysis conducted based on treatment duration.

**Figure 3:**
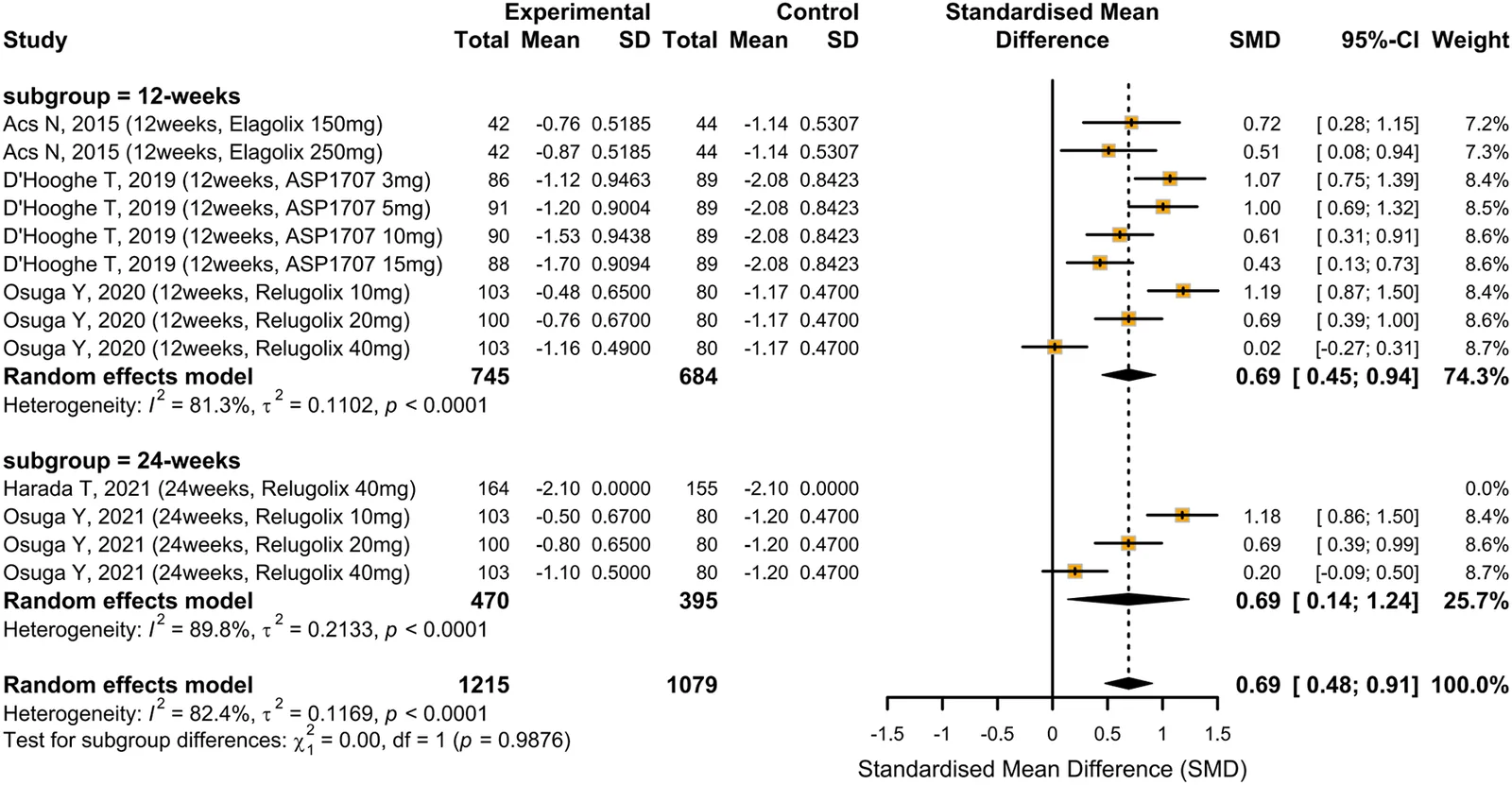
Forest plot of changes from baseline in dysmenorrhea scores, stratified by treatment duration.

#### 3.4.2 Subgroup analysis by type of GnRH-ant

As shown in **Figure 4**, subgroup analysis was performed according to the type of drug administered in the experimental group, with the included studies classified into three subgroups: elagolix, ASP1707, and relugolix. The elagolix subgroup presented a significant between-group difference with an SMD of 0.61 (95% CI: 0.31 to 0.92), and low heterogeneity (I² = 0%, τ² = 0, P = 0.5065). In the ASP1707 subgroup, the pooled SMD was 0.78 (95% CI: 0.47 to 1.08), accompanied by moderate-to-substantial heterogeneity (I² = 74.1%, τ² = 0.0699, P = 0.0089), which may be attributed to variable drug dosages across trials. For the relugolix subgroup, the pooled SMD was 0.66 (95% CI: 0.27 to 1.05), indicating significant efficacy differences and high heterogeneity (I² = 89.7%, τ² = 0.2093, P < 0.0001). Heterogeneity in this group was likely driven by inconsistent dosage regimens and follow-up durations. When all studies were pooled, the overall SMD was 0.69 (95% CI: 0.48 to 0.91), favoring GnRH-a for dysmenorrhea improvement with substantial global heterogeneity (I² = 82.4%, τ² = 0.1169, P < 0.0001). Subgroup difference testing showed no significant discrepancy in effect sizes among the three GnRH-ant subgroups (χ² = 0.58, df = 2, P = 0.7427). In summary, the choice of different GnRH-ant agents did not substantially alter the comparative therapeutic effect on dysmenorrhea relief.

**Figure 4:**
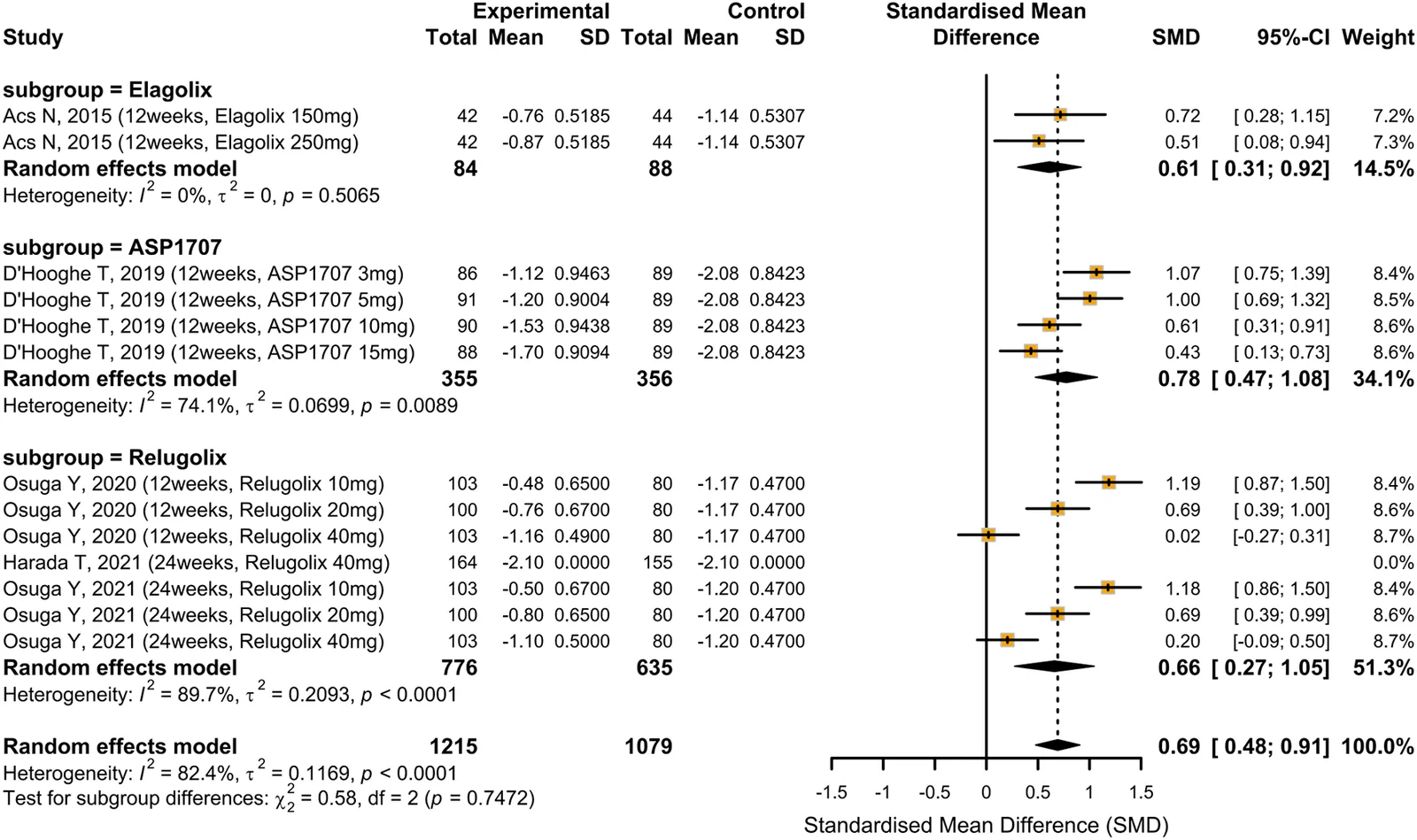
Forest plot of changes from baseline in dysmenorrhea scores, stratified by type of GnRH-ant.

### 3.5 Changes in nonmenstrual pelvic pain

#### 3.5.1 Subgroup analysis by treatment duration

To compare the effects of GnRH-ant and GnRH-a on NMPP, subgroup analysis was performed according to treatment duration. As shown in **Figure 5**, the overall pooled SMD was 0.33 (95% CI: 0.16 to 0.50), demonstrating superior NMPP relief with GnRH-a, with moderate between-study heterogeneity (I² = 53.1%, τ² = 0.0264, P = 0.0463). Leave-one-out sensitivity analysis showed that exclusion of individual studies did not materially alter the pooled estimate, supporting the stability of the findings (**Supplementary Figure 2**). In the 12-week treatment subgroup, the pooled SMD was 0.40 (95% CI: 0.26 to 0.53), favoring GnRH-a with highly consistent results and negligible heterogeneity (I² = 0%, τ² = 0, P = 0.6118). In contrast, the 24-week subgroup included only one study, with an SMD of 0.00 (95% CI: −0.22 to 0.22), suggesting no detectable intergroup difference in NMPP improvement. The test for subgroup differences showed a statistically significant difference in effect estimates between the 12-week and 24-week subgroups (χ² = 9.23, df = 1, P = 0.0024), however, these findings should be interpreted cautiously given potential confounding by antagonist type, dosage, and study design.

**Figure 5:**
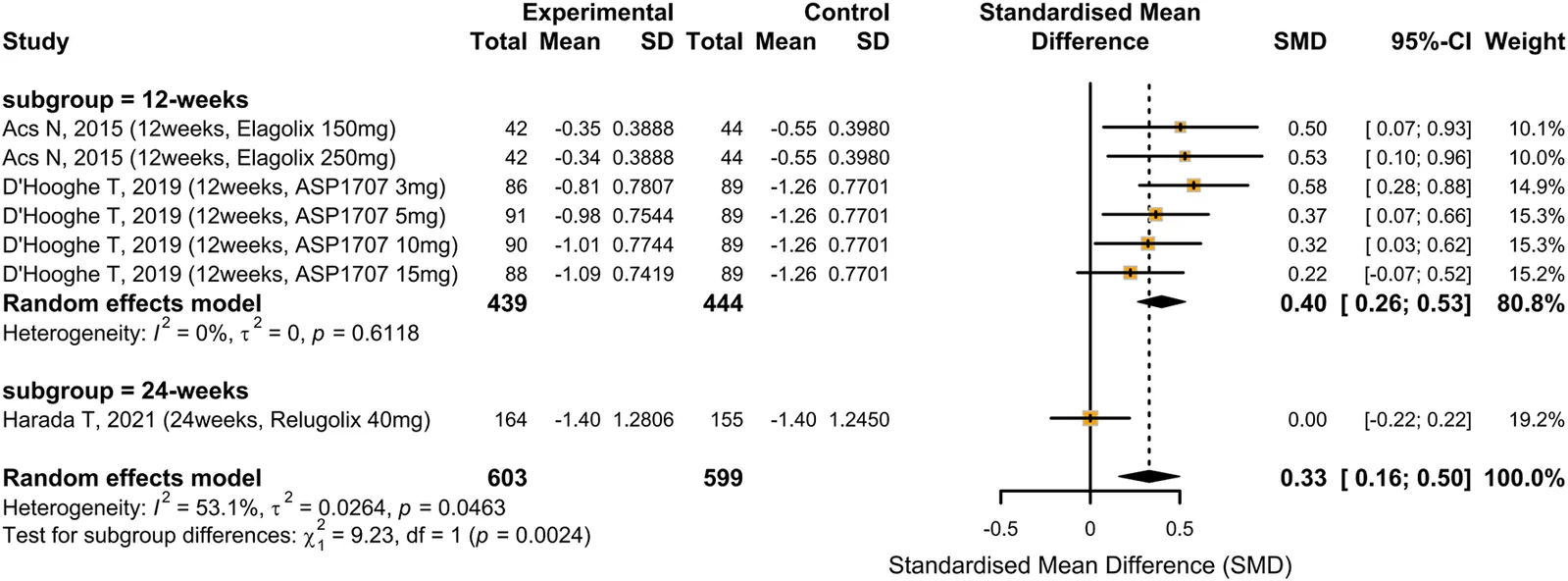
Forest plot of changes from baseline in NMPP scores, stratified by treatment duration.

#### 3.5.2 Subgroup analysis by type of GnRH-ant

We also conducted drug-specific subgroup analysis for NMPP (**Figure 6**). The overall pooled SMD was 0.33 (95% CI: 0.16 to 0.50), favoring GnRH-a for NMPP improvement, with moderate inter-study heterogeneity (I² = 53.1%, τ² = 0.0264, P = 0.0463). Stratified by GnRH-ant agent, the elagolix subgroup produced an SMD of 0.52 (95% CI: 0.21 to 0.82), in favor of GnRH-a, with zero heterogeneity (I² = 0%, τ² = 0, P = 0.9353). A similar pattern was observed in the ASP1707 subgroup, with a pooled SMD of 0.37 (95% CI: 0.22 to 0.52), also favoring GnRH-a and exhibiting no meaningful heterogeneity (I² = 0%, τ² = 0, P = 0.4148). In contrast, the relugolix subgroup yielded an SMD of 0.00 (95% CI: −0.22 to 0.22), demonstrating no detectable intergroup difference. Subgroup-difference testing revealed significant divergence across the three agents (χ² = 9.94, df = 2, P = 0.0069). These findings indicate that the comparative performance of GnRH-ant versus GnRH-a for NMPP varies by antagonist type: elagolix and ASP1707 trials favored GnRH-a, while no treatment gap was seen within the relugolix subgroup.

**Figure 6:**
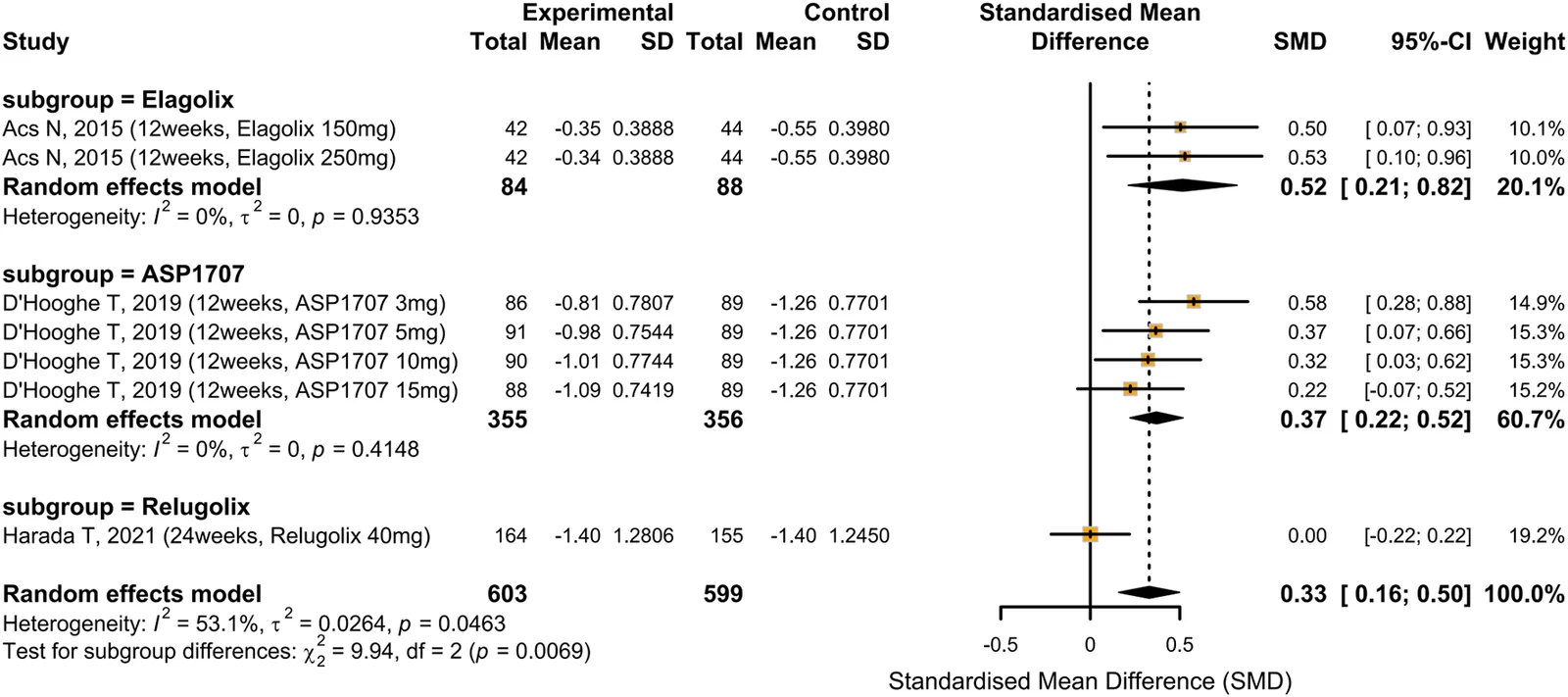
Forest plot of changes from baseline in NMPP scores, stratified by type of GnRH-ant.

### 3.6 Changes in overall pelvic pain

#### 3.6.1 Subgroup analysis by treatment duration

Subgroup analysis stratified by treatment duration were performed to evaluate changes in OPP (**Figure 7**). The overall pooled SMD was 0.25 (95% CI: 0.13 to 0.38), favoring GnRH-a for OPP reduction with negligible inter-study heterogeneity (I² = 0.0%, τ² = 0.0068, P = 0.4925). Leave-one-out sensitivity analysis indicated that the pooled effect remained consistent after sequential exclusion of individual studies (**Supplementary Figure 3**). The 24-week subgroup, containing four effect estimates, produced a pooled SMD of 0.25 (95% CI: 0.09 to 0.42) in favor of GnRH-a and low within-group heterogeneity (I² = 28.7%, τ² = 0.0068, P = 0.2398), indicating relatively good consistency across studies. Similarly, the 12-week subgroup, with two effect estimates, yielded an SMD of 0.30 (95% CI: −0.00 to 0.60), with no substantial heterogeneity observed (I² = 0%, τ² = 0.0068, P = 0.7547). While the point estimate favored GnRH-a, the 95% CI crossed the null value, requiring cautious interpretation. Despite minor divergence in point estimates between subgroups, the test for subgroup differences under the fixed-effect model showed no statistical significance (χ² = 0.10, df = 1, P = 0.7517).

**Figure 7:**
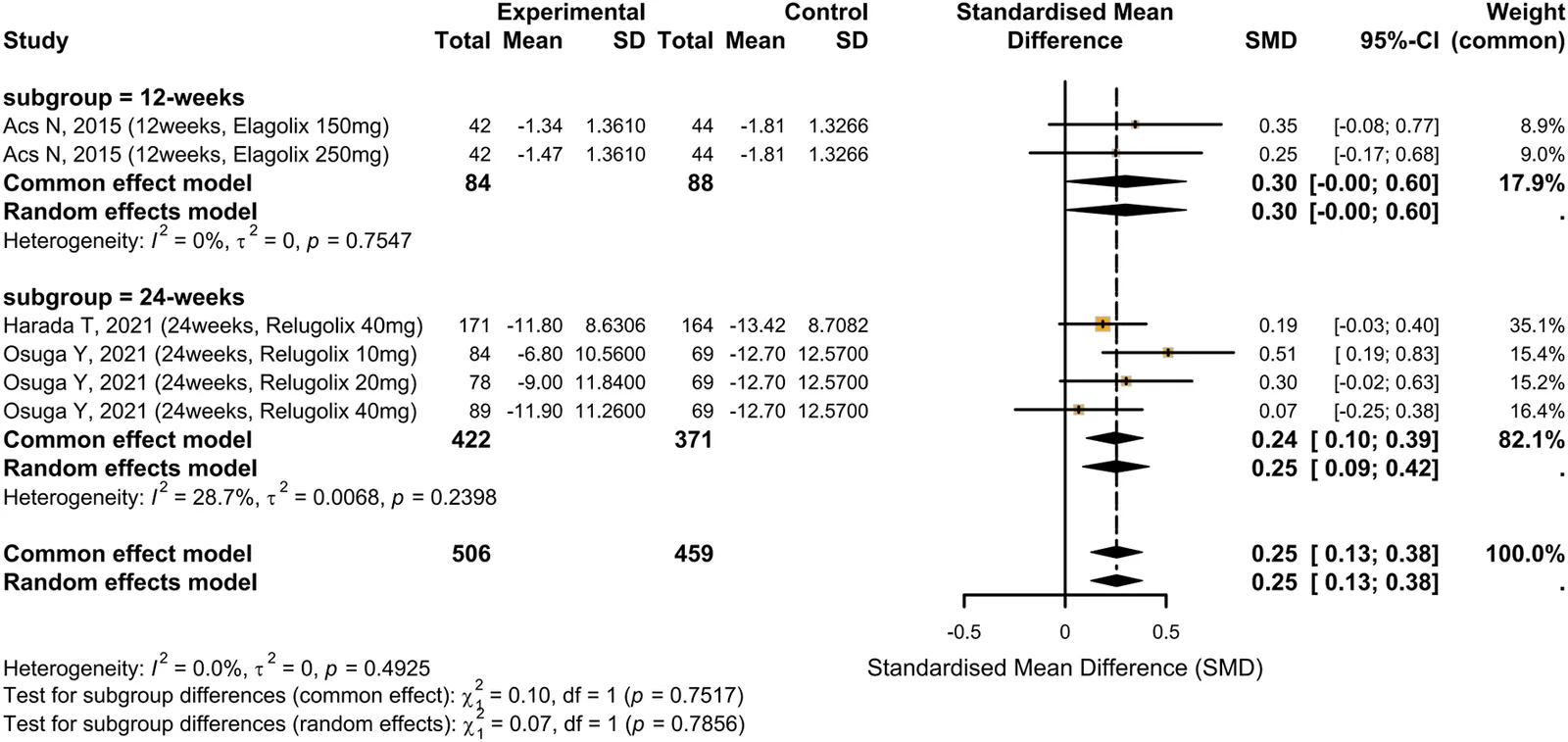
Forest plot of changes from baseline in OPP scores, stratified by treatment duration.

#### 3.6.2 Subgroup analysis by type of GnRH-ant

As shown in **Figure 8**, drug-specific subgroup analysis was performed to compare the efficacy of different GnRH-ant agents versus GnRH-a for improving OPP. The overall SMD was 0.25 (95% CI: 0.13 to 0.38), favoring GnRH-a with no significant inter-study heterogeneity (I² = 0%, τ² = 0, P = 0.4925). The relugolix subgroup included four effect estimates, with a pooled SMD of 0.25 (95% CI: 0.09 to 0.42). GnRH-a demonstrated superior OPP relief, with low within-subgroup heterogeneity (I² = 28.7%, τ² = 0.0068, P = 0.2398). The elagolix subgroup included two effect estimates and produced an SMD of 0.30 (95% CI: −0.00 to 0.60). Although the direction of effect also favored GnRH-a, the confidence interval crossed the null value, indicating no statistically significant intergroup difference, with zero observed heterogeneity (I² = 0%, τ² = 0, P = 0.7547). Subgroup difference testing based on the fixed-effect model revealed no significant discrepancy between relugolix and elagolix subgroups (χ² = 0.10, df = 1, P = 0.7517). Therefore, current evidence suggests comparable relative efficacy of relugolix and elagolix versus GnRH-a in alleviating OPP.

**Figure 8:**
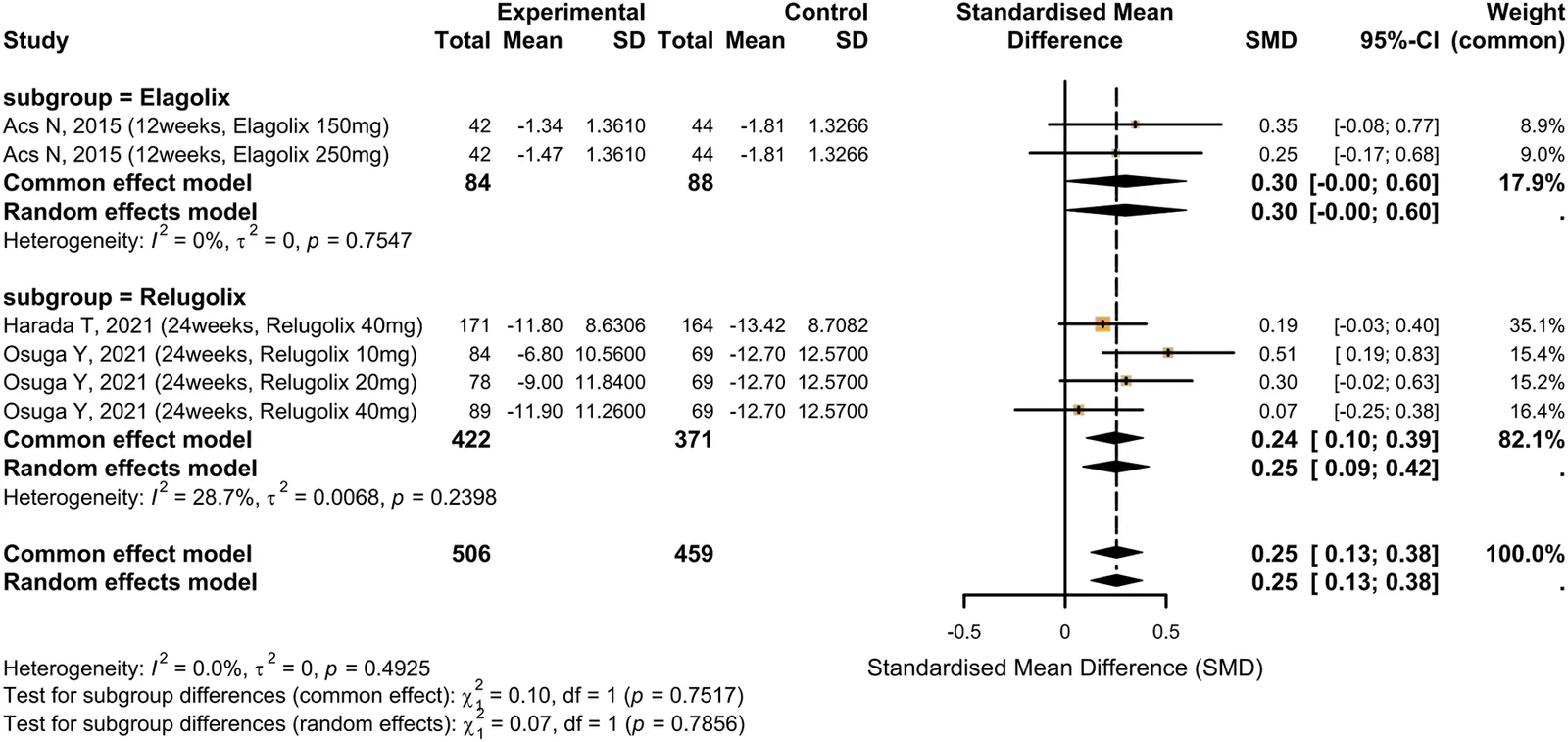
Forest plot of changes from baseline in OPP scores, stratified by type of GnRH-ant.

### 3.7 Changes in dyspareunia

#### 3.7.1 Subgroup analysis by treatment duration

Subgroup analysis stratified by treatment duration assessed changes in dyspareunia scores (**Figure 9**). The 12-week subgroup contained seven effect estimates, with a pooled SMD of 0.17 (95% CI: 0.02 to 0.32) favoring GnRH-a for dyspareunia improvement and low within-group heterogeneity (I² = 28%, τ² = 0.0075, P = 0.2148). The 24-week subgroup included four effect estimates, yielding an SMD of 0.05 (95% CI: −0.22 to 0.32), showing no detectable intergroup difference. The overall SMD was 0.14 (95% CI: 0.02 to 0.26), demonstrating a modest benefit of GnRH-a for dyspareunia relief without substantial inter-study heterogeneity (I² = 0%, τ² = 0, P = 0.5210). Leave-one-out sensitivity analysis demonstrated that the pooled estimate was not substantially affected by the removal of any individual study (**Supplementary Figure 4**). Despite numerical divergence between the two subgroups, the fixed-effect-model test for subgroup differences was non-significant (χ² = 0.48, df = 1, P = 0.4905).

**Figure 9:**
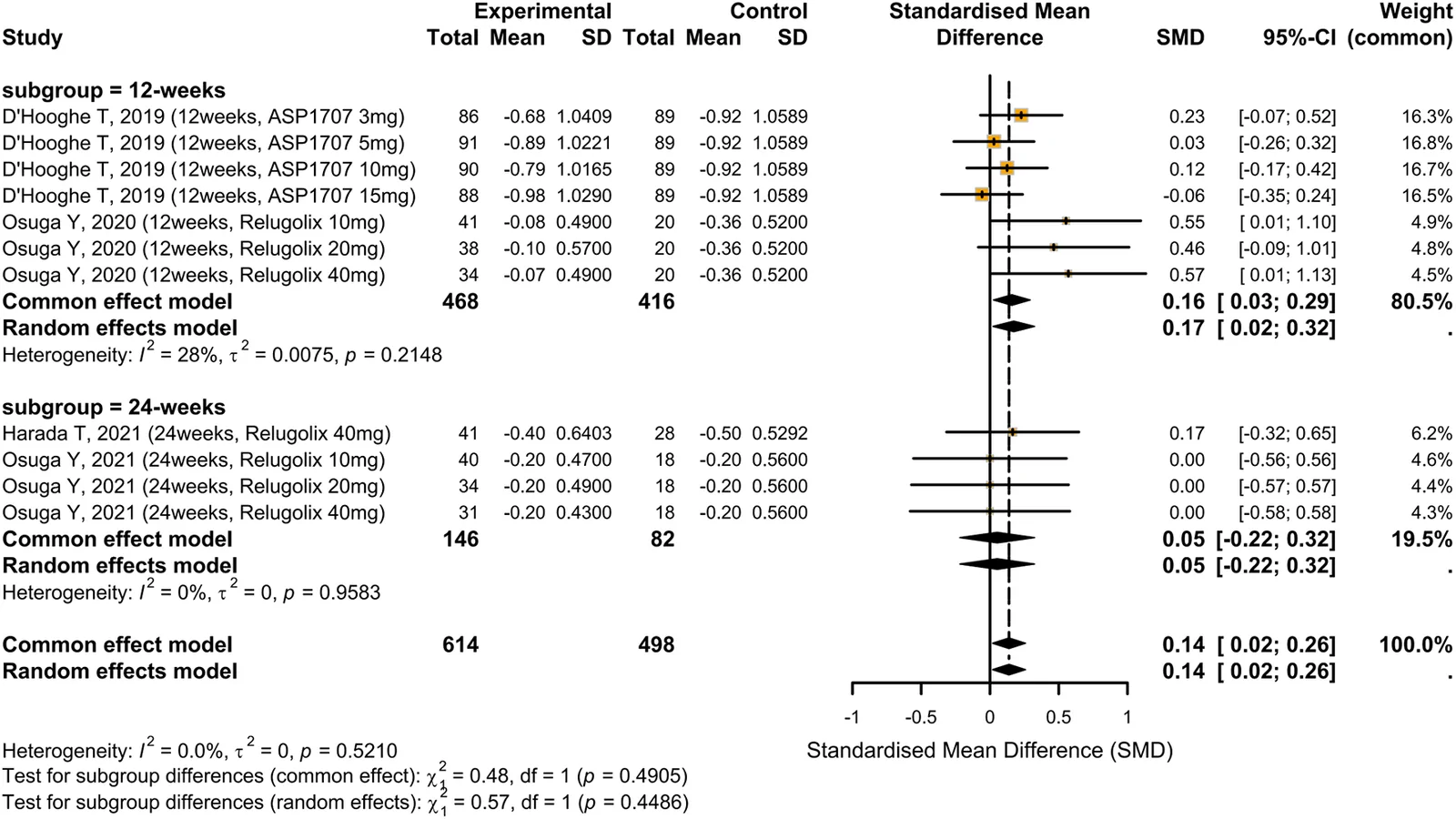
Forest plot of changes from baseline in dyspareunia scores, stratified by treatment duration.

#### 3.7.2 Subgroup analysis by type of GnRH-ant

Drug-based subgroup analysis were conducted for dyspareunia (**Figure 10**). Globally, GnRH-a conferred a modest benefit over GnRH-ant for dyspareunia relief (SMD = 0.14, 95% CI: 0.02 to 0.26), with no substantial heterogeneity observed (I² = 0%, τ² = 0, P = 0.5210). The relugolix subgroup yielded an SMD of 0.25 (95% CI: 0.05 to 0.46), favoring GnRH-a, indicating superior dyspareunia improvement, and zero within-group heterogeneity (I² = 0%, τ² = 0, P = 0.4991). For the ASP1707 subgroup, the SMD was 0.08 (95% CI: −0.07 to 0.23). Despite a point estimate leaning towards GnRH-a, the confidence interval crossed the null value, showing no statistically significant intergroup difference (I² = 0%, τ² = 0, P = 0.5752). Subgroup interaction testing detected no statistical discrepancy between relugolix and ASP1707 (χ² = 1.78, df = 1, P = 0.1827). Existing evidence cannot confirm divergent comparative effects of GnRH-a relative to relugolix and ASP1707 on dyspareunia improvement.

**Figure 10:**
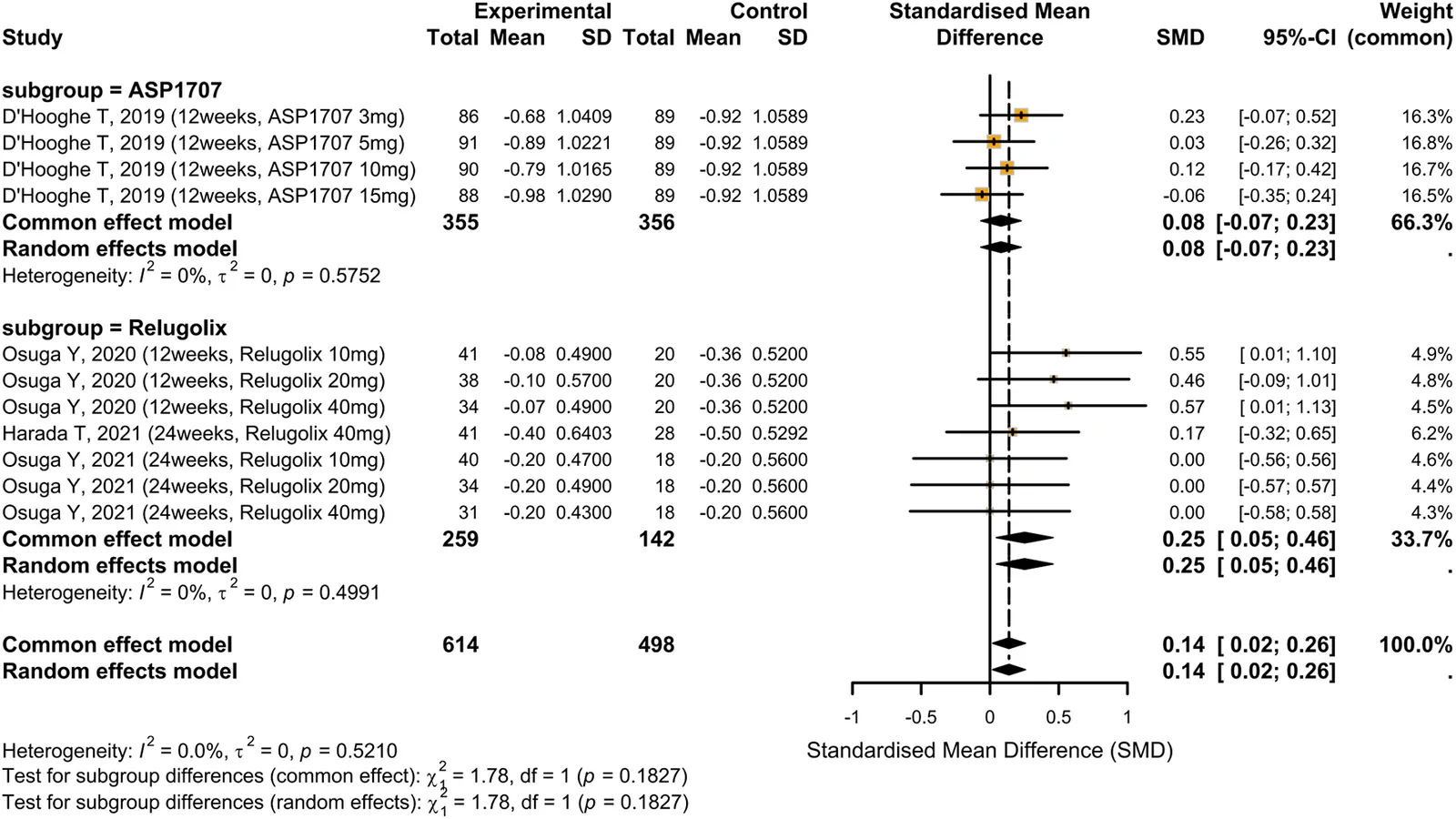
Forest plot of changes from baseline in dyspareunia scores, stratified by type of GnRH-ant.

### 3.8 Changes in pelvic tenderness

#### 3.8.1 Subgroup analysis by treatment duration

We conducted subgroup analysis according to treatment duration, as shown in **Figure 11**, to explore its impact on comparative efficacy for pelvic tenderness. GnRH-a showed a modest advantage over GnRH-ant in reducing pelvic tenderness (SMD = 0.22, 95% CI: 0.07 to 0.37), accompanied by substantial overall heterogeneity (I² = 64.0%, τ² = 0.0384, P = 0.0020). Leave-one-out sensitivity analysis confirmed the stability of the pooled estimate, with no meaningful changes observed after exclusion of individual studies (**Supplementary Figure 5**). The 12-week subgroup presented a statistically significant effect with moderate heterogeneity (SMD = 0.24, 95% CI: 0.06 to 0.42, I² = 60.6%, τ² = 0.0359, P = 0.0185). By contrast, the 24-week subgroup showed no detectable intergroup difference and high within-group heterogeneity (SMD = 0.20, 95% CI: −0.08 to 0.48, I² = 73.4%, τ² = 0.0580, P = 0.0103). Despite significance being attained only in the 12-week stratum, the test for subgroup differences revealed no divergence in effect sizes (χ² = 0.05, df = 1, P = 0.8155).

**Figure 11:**
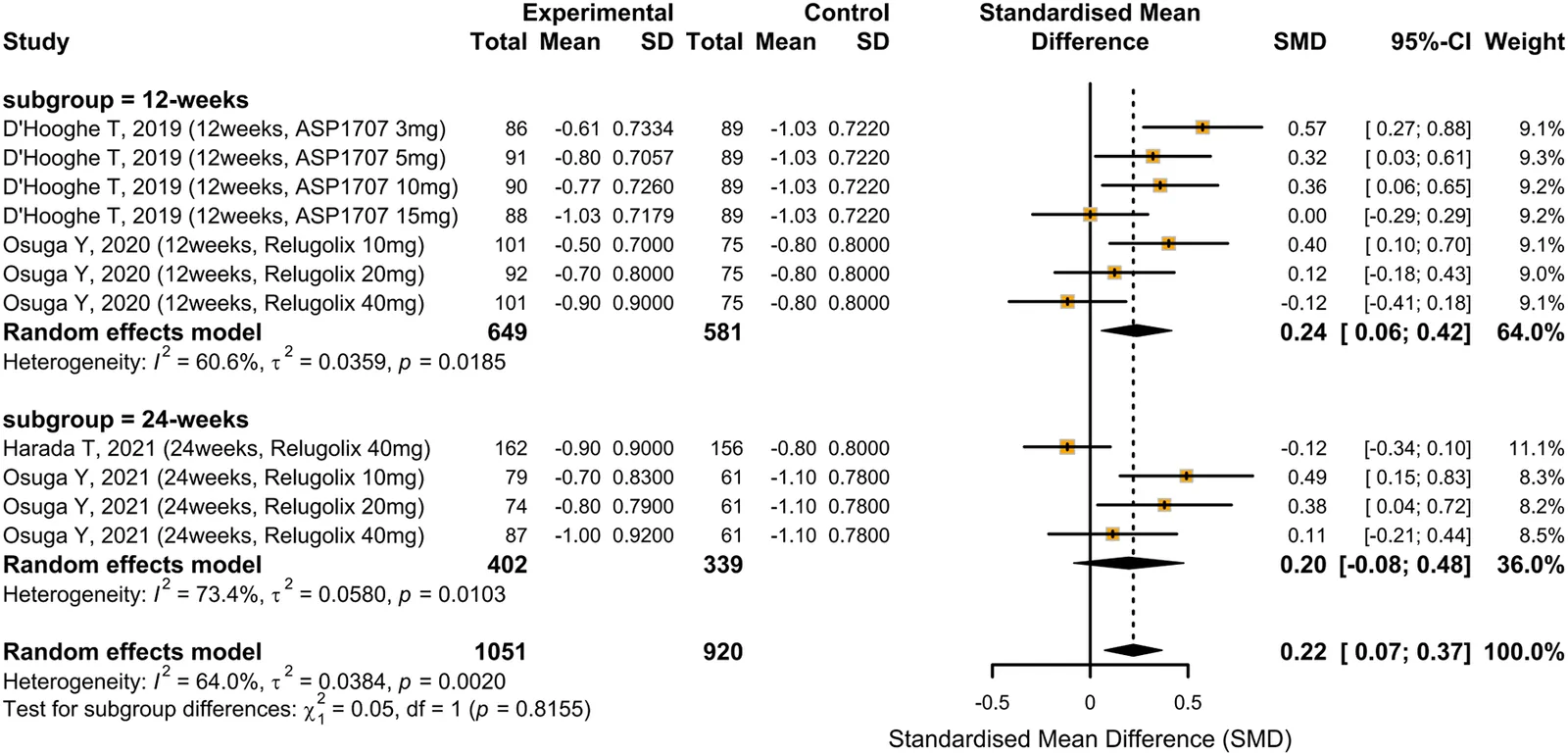
Forest plot of changes from baseline in pelvic tenderness scores, stratified by treatment duration.

#### 3.8.2 Subgroup analysis by type of GnRH-ant

Drug-specific subgroup analysis are presented in **Figure 12**. The overall SMD was 0.22 (95% CI: 0.07 to 0.37), showing a statistically significant modest benefit of GnRH-a, alongside substantial inter-study heterogeneity (I² = 64.0%, τ² = 0.0384, P = 0.0020). The ASP1707 subgroup yielded a significant pooled effect (SMD = 0.31, 95% CI: 0.08 to 0.54, I² = 58.9%, τ² = 0.0329, P = 0.0630), favoring GnRH-a over ASP1707 for pelvic-tenderness relief. No significant intergroup difference was detected within the relugolix subgroup (SMD = 0.17, 95% CI: −0.02 to 0.36, I² = 64.7%, τ² = 0.0406, P = 0.0094). Although the effect estimates differed numerically between the ASP1707 and relugolix subgroups, the test for subgroup differences was not statistically significant (χ² = 0.90, df = 1, P = 0.3434). Existing evidence cannot confirm divergent relative efficacy of ASP1707 and relugolix versus GnRH-a on pelvic tenderness.

**Figure 12:**
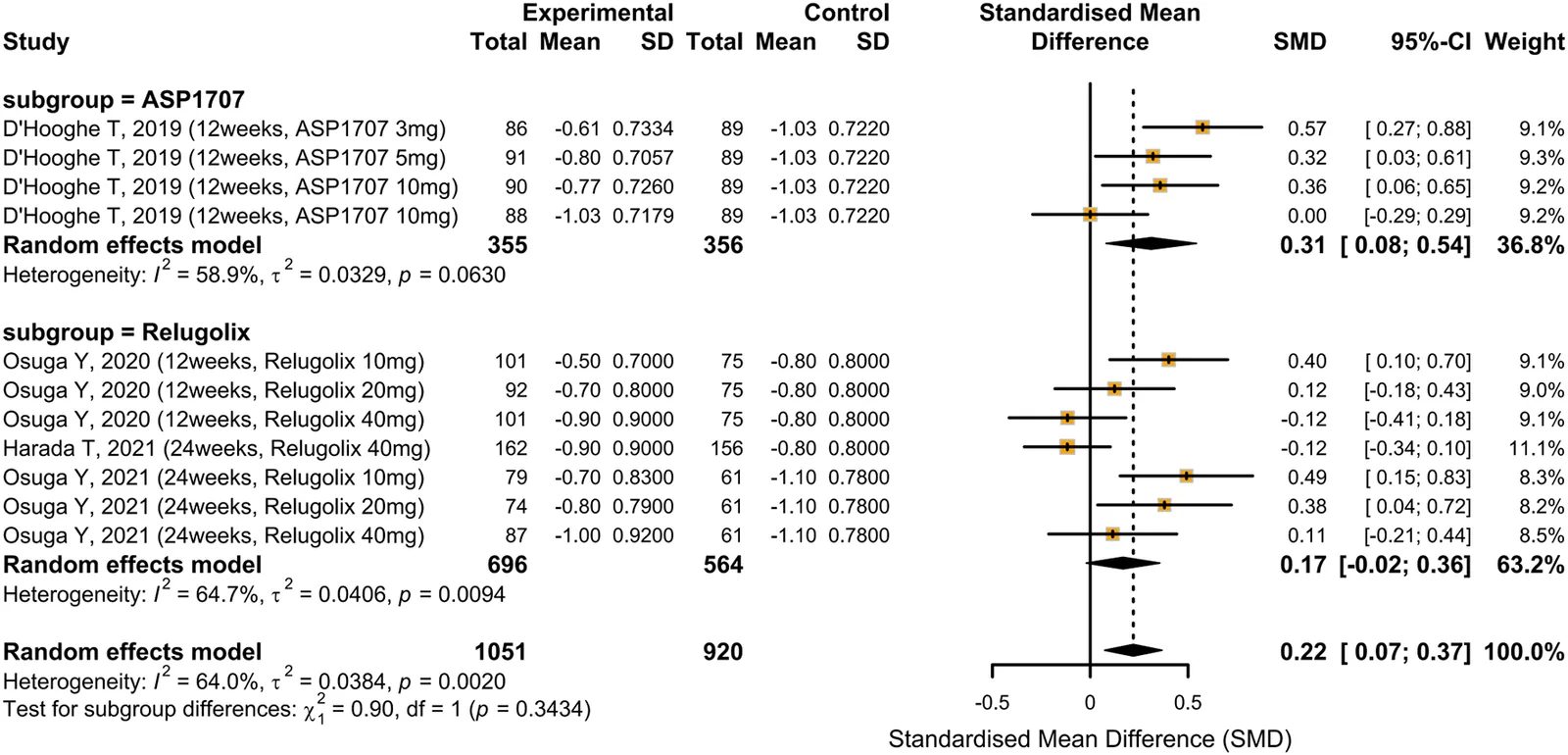
Forest plot of changes from baseline in pelvic tenderness scores, stratified by type of GnRH-ant.

### 3.9 Changes in BMD

To further assess whether treatment duration and GnRH-ant agent type influenced BMD, subgroup analysis was performed accordingly. The overall pooled result clearly favored the GnRH-ant (SMD = 0.64, 95% CI: 0.39 to 0.89, P < 0.0001), indicating that GnRH-ant as a class were produced less BMD reduction than GnRH-a, with substantial inter-study heterogeneity (I² = 79.4%, τ² = 0.1173, P < 0.0001). Leave-one-out sensitivity analysis indicated that the pooled estimate remained stable after sequential exclusion of individual studies (**Supplementary Figure 6**). When stratified by treatment duration, as shown in **Figure 13**, the results favored GnRH-ant in both the 12-week subgroup (SMD = 0.37, 95% CI: 0.15 to 0.60, I² = 55.3%, τ² = 0.0356, P = 0.0622) and the 24-week subgroup (SMD = 0.98, 95% CI: 0.76 to 1.20, I² = 30.2%, τ² = 0.0152, P = 0.2313). Subgroup difference testing was statistically significant (χ² = 14.44, df = 1, P = 0.0001), with a larger effect in the 24-week subgroup. Drug-specific subgroup analysis (**Figure 14**) showed effects favoring GnRH-ant across all three agents. The largest effect estimate was observed in the ASP1707 subgroup (SMD = 0.98, 95% CI: 0.76 to 1.20, I² = 30.2%, τ² = 0.0152, P = 0.2313), followed by the relugolix subgroup (SMD = 0.39, 95% CI: 0.04 to 0.75, I² = 77.1%, τ² = 0.0768, P = 0.0126) and elagolix (SMD = 0.33, 95% CI: 0.02 to 0.63, I² = 0%, τ² = 0, P = 0.7736). The inter-subgroup difference was significant (χ² = 15.01, df = 2, P = 0.0005), indicating that the magnitude of BMD-sparing effect varies among GnRH-ant agents, with ASP1707 demonstrating the most pronounced advantage.

**Figure 13:**
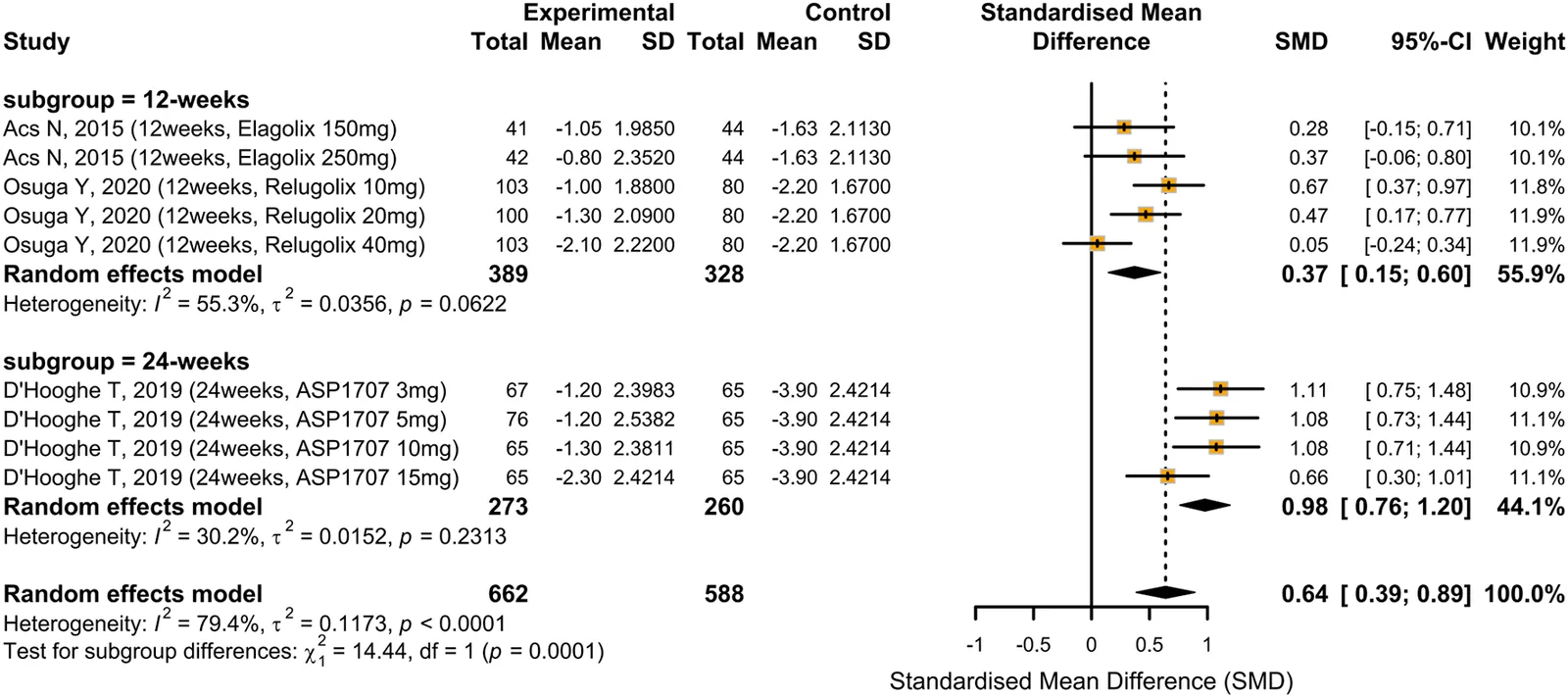
Forest plot of changes from baseline in BMD, stratified by treatment duration.

**Figure 14:**
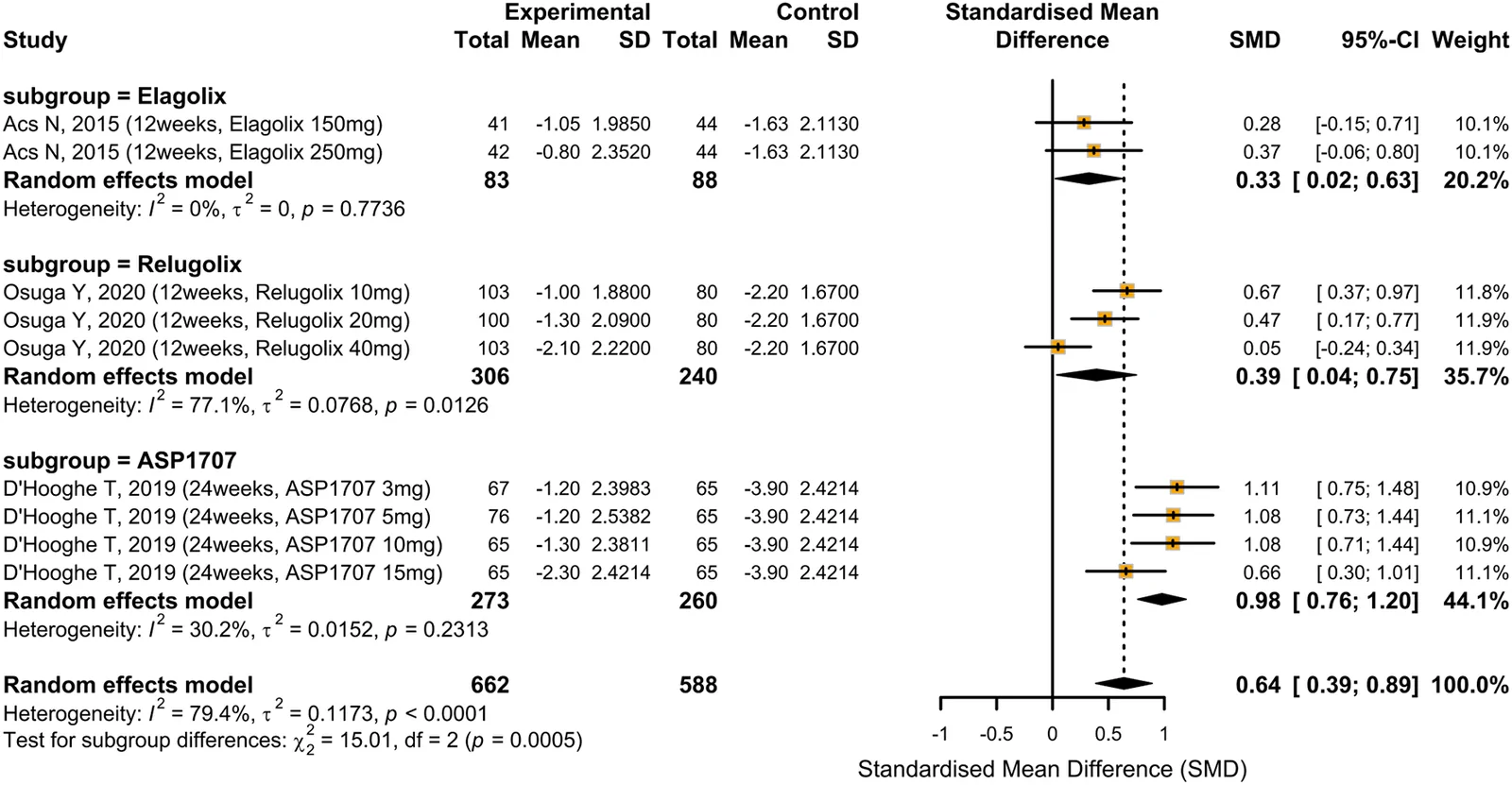
Forest plot of changes from baseline in BMD, stratified by type of GnRH-ant.

### 3.10 Changes in induration of the Douglas cavity score

#### 3.10.1 Subgroup analysis by treatment duration

In addition, subgroup analysis stratified by treatment duration assessed changes in induration of the Douglas cavity score (**Supplementary Figure 7**). The overall pooled result revealed a significant difference favoring GnRH-a (SMD = 0.15, 95% CI: 0.07 to 0.24), with no substantial heterogeneity observed (I² = 0.0%, τ² = 0, P = 0.8983). The 12-week subgroup showed a statistically significant benefit for GnRH-a (SMD = 0.17, 95% CI: 0.06 to 0.29, I² = 0%, τ² = 0, P = 0.7393), with the effect again favoring GnRH-a, whereas no significant intergroup difference was detected in the 24-week subgroup (SMD = 0.12, 95% CI: −0.02 to 0.27, I² = 0%, τ² = 0, P = 0.7903). The test for subgroup differences was not statistically significant (χ² = 0.31, df = 1, P = 0.5769). Current evidence does not identify divergent comparative effects between 12-week and 24-week regimens for the Douglas cavity induration improvement.

#### 3.10.2 Subgroup analysis by type of GnRH-ant

Drug-specific subgroup analysis are summarised in **Supplementary Figure 8**. The overall between-group difference was statistically significant, with the effect favoring GnRH-a (SMD = 0.15, 95% CI: 0.07 to 0.24), with no heterogeneity observed (I² = 0.0%, τ² = 0, P = 0.8983). Both the ASP1707 subgroup (SMD = 0.20, 95% CI: 0.06 to 0.35, I² = 0%, τ² = 0, P = 0.6623) and the relugolix subgroups (SMD = 0.13, 95% CI: 0.01 to 0.24, I² = 0%, τ² = 0, P = 0.8586) yielded significant effects in favor of GnRH-a. Subgroup-difference testing revealed no statistical discrepancy between the two antagonists (χ² = 0.72, df = 1, P = 0.3927). Current evidence cannot confirm divergent relative effects of ASP1707 and relugolix versus GnRH-a for improving induration of the Douglas cavity.

### 3.11 Changes in peak score for endometriosis pain

We performed subgroup analysis according to treatment duration and the specific type of GnRH-ant to compare the effects of GnRH-ant and GnRH-a on endometriosis pain peak scores (**Supplementary Figures 9**, **10**). The overall pooled analysis identified a significant difference favoring GnRH-a (SMD = 0.36, 95% CI: 0.18 to 0.54), with no substantial inter-study heterogeneity (I² = 0.0%, τ² = 0.0043, P = 0.3712). When stratified by treatment duration, both the 12-week subgroup (SMD = 0.52, 95% CI: 0.22 to 0.82, I² = 0%, τ² = 0, P = 0.5322) and the 24-week subgroup (SMD = 0.28, 95% CI: 0.06 to 0.50) showed effects favoring GnRH-a. Subgroup difference testing confirmed no statistical discrepancy in effect sizes between the two durations (χ² = 1.59, df = 1, P = 0.2071). Consistent results were observed in drug-type stratification. Both the elagolix subgroup (SMD = 0.52, 95% CI: 0.22 to 0.82, I² = 0%, τ² = 0, P = 0.5322) and the relugolix subgroup (SMD = 0.28, 95% CI: 0.06 to 0.50) favored GnRH-a. The test for subgroup differences was again not statistically significant (χ² = 1.59, df = 1, P = 0.2071). Therefore, current evidence suggests comparable relative efficacy of elagolix and relugolix versus GnRH-a in reducing endometriosis peak pain scores.

### 3.12 Changes in the percentage of days of analgesic use

For the analysis of the percentage of days of analgesic medications used, subgroup analysis was performed according to treatment duration and the specific GnRH-ant agent type (**Supplementary Figures 11**, **12**). The overall pooled results indicated that GnRH-a significantly reduced analgesic consumption days compared with GnRH-ant (SMD = 0.16, 95% CI: 0.06 to 0.26). Only low heterogeneity was observed across the included studies (I² = 0.0%, τ² = 0.0010, P = 0.3522). When stratified by treatment duration, the between-group difference was statistically significant in the 24-week subgroup and favored GnRH-a (SMD = 0.20, 95% CI: 0.06 to 0.35, I² = 0%, τ² < 0.0001, P = 0.4058). By comparison, the 12-week subgroup showed no statistically significant intergroup difference (SMD = 0.12, 95% CI: −0.03 to 0.27, I² = 25.6%, τ² = 0.0093, P = 0.2511). Despite numerical differences in effect sizes, fixed-effect subgroup comparison detected no significant discrepancy between the two durations (χ² = 0.60, df = 1, P = 0.4380). Drug-specific stratification revealed significant benefits of GnRH-a in both the elagolix subgroup (SMD = 0.31, 95% CI: 0.01 to 0.61, I² = 0%, τ² = 0, P = 0.3379) and the relugolix subgroup (SMD = 0.14, 95% CI: 0.03 to 0.25, I² = 12.9%, τ² = 0.0004, P = 0.3313). No statistical difference was found between the two antagonist subgroups (χ² = 1.08, df = 1, P = 0.2992). Collectively, current evidence demonstrates that neither treatment duration nor GnRH-ant agent type significantly modifies the comparative efficacy of GnRH-a versus GnRH-ant in reducing analgesic usage frequency. However, considering various confounding factors, the interpretation of this result should be cautious.

### 3.13 Changes in menstrual blood loss

We also performed subgroup analysis of changes from baseline in menstrual blood loss. As shown in **Supplementary Figures 13** and **14**, the overall pooled effect favored GnRH-a with statistical significance (SMD = 0.77, 95% CI: 0.05 to 1.50). Nevertheless, substantial inter-study heterogeneity was present (I² = 95.6%, τ² = 0.6949, P < 0.0001), indicating the result requires cautious interpretation. Leave-one-out sensitivity analysis showed that the direction of the pooled effect remained unchanged after exclusion of individual studies, although some variation in effect magnitude was observed (**Supplementary Figure 15**). Stratification by treatment duration showed a significant benefit of GnRH-a within the 24-week subgroup (SMD = 0.36, 95% CI: 0.14 to 0.58). No significant intergroup difference was observed in the 12-week subgroup (SMD = 0.92, 95% CI: −0.04 to 1.88, I² = 96.5%, τ² = 0.5303, P < 0.0001). The test for subgroup differences was not statistically significant (χ² = 1.23, df = 1, P = 0.2681). When stratified by the type of GnRH-ant, all included studies for this outcome investigated relugolix. Hence, drug-type subgroup comparison was not feasible. The pooled estimate for relugolix matched the overall result (SMD = 0.77, 95% CI: 0.05 to 1.50, I² = 95.6%, τ² = 0.5303, P < 0.0001) and reached statistical significance. Marked heterogeneity mandates cautious interpretation of this finding.

### 3.14 Assessment of quality of life in patients with endometriosis

Four included studies evaluated patient quality of life using the EHP-5 or EHP-30 questionnaire, encompassing five domains: Pain, Control and Powerlessness, Emotional Wellbeing, Social Support, and Self-image. Subgroup analysis stratified by treatment duration and GnRH-ant agent type were performed for each domain to compare the effects of GnRH-ant and GnRH-a on quality of life.

#### 3.14.1 Subgroup analysis by treatment duration

For the five quality-of-life domains, subgroup analysis were first performed according to treatment duration (**Supplementary Figures 16**-**20**). For the Pain domain (**Supplementary Figure 16**), fixed-effect pooling yielded an SMD of 0.21 (95% CI: 0.11 to 0.31), favoring GnRH-a with low heterogeneity (I² = 14.6%, τ² = 0.0033, P = 0.3119). Both the 12-week subgroup (SMD = 0.19, 95% CI: 0.04 to 0.34, I² = 30.5%, τ² = 0.0144, P = 0.2182) and the 24-week (SMD = 0.23, 95% CI: 0.08 to 0.37, I² = 14.9%, τ² < 0.0001, P = 0.3173) favored GnRH-a, with no significant inter-subgroup difference (χ² = 0.09, df = 1, P = 0.7632).

The Control and Powerlessness domain (**Supplementary Figure 17**) showed a similar pattern, with an overall SMD of 0.22 (95% CI: 0.11 to 0.32), and no substantial heterogeneity was observed (I² = 0.0%, τ² = 0, P = 0.8074). Statistically significant between-group differences were observed in both the 12-week subgroup (SMD = 0.19, 95% CI: 0.04 to 0.34) and the 24-week (SMD = 0.24, 95% CI: 0.09 to 0.38), with no significant subgroup difference (χ² = 0.18, df = 1, P = 0.6682).

In contrast, no significant between-group differences were identified for the Emotional Wellbeing, Social Support, or Self-image domains. Emotional Wellbeing (**Supplementary Figure 18**) yielded an overall SMD of −0.01 (95% CI: −0.11 to 0.09, I² = 0.0%). The 95% CIs for both the 12-week subgroup (SMD = 0.00, 95% CI: −0.15 to 0.15) and the 24-week subgroup (SMD = −0.02, 95% CI: −0.17 to 0.12) crossed the null value, and the difference between subgroups was not statistically significant (χ² = 0.07, df = 1, P = 0.7913). Social Support domain (**Supplementary Figure 19**) showed an identical overall SMD of −0.01 (95% CI: −0.11 to 0.09, I² = 0.0%, τ² = 0, P = 0.9381). Neither the 12-week subgroup (SMD = −0.03) nor the 24-week subgroup (SMD = 0.01) showed a clear effect, and no significant difference was observed between the treatment-duration subgroups (χ² = 0.19, df = 1, P = 0.6631). Self-image (**Supplementary Figure 20**) also remained near zero (SMD = −0.01, 95% CI: −0.11 to 0.10, I² = 0.0%, τ² = 0, P = 0.8586). with identical estimates in both subgroups (SMD = −0.01 each; χ² = 0.00, df = 1, P = 0.9816).

#### 3.14.2 Subgroup analysis by type of GnRH-ant

Drug-specific subgroup analysis were further performed across the five quality-of-life domains (**Supplementary Figures 21**-**25**). For the Pain domain (**Supplementary Figure 21**), the overall pooled SMD was 0.21 (95% CI: 0.11 to 0.31), favoring GnRH-a with low heterogeneity (I² = 14.6%, τ² = 0.0033, P = 0.3119). Both elagolix (SMD = 0.36, 95% CI: 0.06 to 0.66, I² = 0%, τ² = 0, P = 0.4216) and relugolix subgroups (SMD = 0.19, 95% CI: 0.08 to 0.30, I² = 21.7%, τ² = 0.0043, P = 0.2642) favored GnRH-a, with no significant inter-subgroup difference (χ² = 1.07, df = 1, P = 0.3014).

The Control and Powerlessness domain (**Supplementary Figure 22**) also favored GnRH-a (SMD = 0.22, 95% CI: 0.11 to 0.32, I² = 0.0%, τ² = 0, P = 0.8074). The relugolix subgroup reached statistical significance (SMD = 0.23, 95% CI: 0.11 to 0.34, I² = 0%, τ² = 0, P = 0.6572), whereas the elagolix subgroup did not (SMD = 0.15, 95% CI: −0.15 to 0.45, I² = 0%, τ² = 0, P = 0.7096), though the subgroup difference was non-significant (χ² = 0.24, df = 1, P = 0.6263).

No significant between-group differences were detected for the remaining three domains. Emotional Wellbeing (**Supplementary Figure 23**), yielded an overall SMD of −0.01 (95% CI: −0.11 to 0.09, I² = 0.0%, τ² = 0, P = 0.8779), with negligible effects in both elagolix (SMD = 0.06) and relugolix (SMD = −0.02; χ² = 0.28, df = 1, P = 0.5967). For the Social Support domain (**Supplementary Figure 24**), the overall pooled SMD was −0.01 (95% CI: −0.11 to 0.09, I² = 0.0%, τ² = 0, P = 0.9381), with no clear effect in either subgroup (elagolix: SMD = −0.06; relugolix: SMD = −0.00; P for subgroup difference = 0.7310, χ² = 0.12, df = 1). Self-image domain (**Supplementary Figure 25**) also remained near zero (SMD = −0.01, 95% CI: −0.11 to 0.10, I² = 0.0%, τ² = 0, P = 0.8586), with comparable small estimates across subgroups (elagolix: SMD = 0.06; relugolix: SMD = −0.02; χ² = 0.23, df = 1, P = 0.6342).

### 3.15 Adverse events and safety analysis

Safety outcomes were reported in all included studies. Across trials, the most frequently observed treatment-emergent adverse events (TEAEs) were mainly related to hypoestrogenic effects, including hot flush, menstrual disturbances (such as metrorrhagia, menorrhagia, and irregular menstruation), and headache. In the Elagolix trial, headache was the most commonly reported adverse event (11). In the Relugolix studies, hot flush and menstrual-related events represented the predominant adverse events (13, 16). In the phase III trial directly comparing Relugolix with leuprolide, treatment discontinuation due to adverse events occurred in 2.9% of patients receiving Relugolix and 4.3% of those receiving leuprolide (14).

Regarding hypoestrogenic effects, all GnRH-based treatment regimens included in this review resulted in suppression of estradiol levels and reductions in BMD to varying degrees. However, the extent of BMD decline was generally smaller with GnRH-ant than with GnRH-a. In the Elagolix study, the reduction in BMD showed a dose-dependent pattern, with mean lumbar spine BMD changes at week 12 of −1.05%, −0.80%, and −1.63% in the Elagolix 150 mg, Elagolix 250 mg, and leuprolide groups (11). Similarly, in the Relugolix extension study, the mean changes from baseline at week 24 were −1.6%, −2.6%, and −4.9% for the Relugolix 10 mg, 20 mg, and 40 mg groups, respectively, compared with −4.4% in the leuprolide group (16). The TERRA trial also demonstrated a dose-dependent reduction in estradiol levels and BMD with ASP1707 treatment (12).

Taken together, both GnRH-ant and GnRH-a showed acceptable short-term tolerability profiles in women with endometriosis-associated pain. However, differences in the degree of estrogen suppression may contribute to variations in hypoestrogenic effects, particularly BMD changes, between these two treatment classes.

## 4 Discussion

This systematic review and meta-analysis comprehensively compared the therapeutic effects of GnRH-ant and leuprolide-based GnRH-a on endometriosis-related symptoms. Current evidence indicates that leuprolide-based GnRH-a confers an advantage across most pain-related outcomes, including dysmenorrhea, NMPP, OPP, dyspareunia, pelvic tenderness, induration of the Douglas cavity, peak score for endometriosis pain, and the percentage of days of analgesic use. Among these outcomes, dysmenorrhea showed the most pronounced between-group difference. For most remaining outcomes, although statistical significance was reached, effect sizes were generally small. Several outcomes showed substantial heterogeneity, particularly dysmenorrhea, menstrual blood loss, pelvic tenderness, and BMD. Subgroup analyses based on treatment duration and GnRH-ant type partially explored potential sources of heterogeneity; however, residual heterogeneity remained, suggesting that variations in drug dosage, treatment duration, baseline symptom severity, and outcome assessment methods may contribute to the observed differences. Therefore, the pooled estimates for highly heterogeneous outcomes, especially menstrual blood loss (I² = 95.6%), should be interpreted with caution. Therefore their clinical relevance warrants careful interpretation within the specific outcome context.

In contrast to the pain-related outcomes, the findings for BMD favored GnRH-ant, with the difference amplifying as treatment duration extended. Meanwhile, no significant between-group difference was observed for the two regimens showed comparable effects on the Emotional Wellbeing, Social Support, and Self-image domains of quality of life. However, it is worth noting that the reason why this study takes GnRH-ant as a class of drugs for research is based on the fact that all of them exert their effects by competitively blocking GnRH receptors, while there are differences in the pharmacokinetic characteristics, administration regimens and the degree of ovarian inhibition among different drugs. Therefore, the pooled analysis results for this class of drugs should be interpreted with caution, as the clinically relevant differences between specific antagonists may not be fully covered.

Although substantial heterogeneity was observed for some outcomes, leave-one-out sensitivity analyses demonstrated that the pooled estimates were generally not driven by any single study. For menstrual blood loss, the direction of effect remained consistent, although variations in effect magnitude were observed after exclusion of individual studies, suggesting that differences in treatment regimens and study characteristics may contribute to the observed heterogeneity.

Collectively, the evidence does not support an overall superiority of one drug class over the other. Rather, the available evidence indicates that the two therapeutic strategies may have distinct efficacy and safety profiles. Therefore, clinical treatment selection should therefore individualise the balance between desired symptom suppression intensity and the risk of hypoestrogenism-related adverse effects.

### 4.1 Interpretation of pain-related efficacy

The present meta-analysis demonstrated greater dysmenorrhea improvement with leuprolide-based GnRH-a, albeit with substantial heterogeneity. The current findings are more appropriately interpreted as indicating an overall trend toward greater improvement in dysmenorrhea with leuprolide-based GnRH-a, rather than as evidence that all GnRH-a are superior to all GnRH-ant for the treatment of dysmenorrhea.

For NMPP, the pooled results likewise favored GnRH-a, although the magnitude of the between-group effect was relatively small. Further analysis according to treatment duration showed a modest advantage of GnRH-a in the 12-week subgroup. The findings were also not entirely consistent across individual GnRH-ant, with differences observed between elagolix or ASP1707 and leuprolide. The findings of the TERRA trial were broadly consistent with this trend: at 12 week, the reduction in NMPP scores was numerically greater in the leuprolide group than in the various ASP1707 dose groups (12). For relugolix, the dose-finding trial conducted by Osuga et al. demonstrated a dose-dependent improvement in pain symptoms, with the efficacy and safety of the 40-mg dose being broadly comparable to those of leuprolide (13, 16). Building on these findings, a phase III randomized non-inferiority trial conducted by Harada et al. further demonstrated that relugolix was non-inferior to leuprolide in improving endometriosis-associated pelvic pain (14). Taken together, these findings suggest that treatment effects may vary among individual GnRH-ant and that their efficacy should not be generalized solely on the basis of drug class.

The differences between the 12-week and 24-week subgroups should be interpreted cautiously, as the current evidence is insufficient to determine whether treatment duration modifies the relative efficacy of GnRH-ant and GnRH-a. The 24-week subgroup drew from a limited number of trials and GnRH-ant regimens, and duration was frequently confounded with drug type, dosage, and study design, precluding isolation of its independent effect. Observed subgroup differences may therefore partly reflect study-level heterogeneity rather than a genuine duration-dependent effect. Current findings suggest that certain GnRH-ant regimens—particularly at lower doses or during earlier treatment phases—may afford less pain relief than leuprolide, though this gap may narrow with prolonged treatment or higher doses. This hypothesis requires confirmation in adequately powered trials and individual participant data meta- analysis.

The pooled effects for OPP, dyspareunia, pelvic tenderness, induration of the Douglas cavity, and peak endometriosis pain were generally (SMD 0.14–0.36). Although some comparisons reached statistical significance, the magnitude of these differences was limited, and they should not be interpreted as evidence of a clinically meaningful superiority of GnRH-a. In addition, pain outcomes were assessed using different scales, including VAS, NRS, B&B, and M-B&B scores. Although SMDs allow quantitative synthesis across different instruments, they are less directly interpretable in terms of patient-perceived improvement. As minimal clinically important differences for these scales were not available for direct comparison, the current evidence supports only a small-to-moderate statistical advantage of GnRH-a for certain pain outcomes, while the clinical relevance of these differences remains uncertain.

The above findings should not be interpreted as evidence against the analgesic efficacy of GnRH-ant. A randomized trial conducted by Ács et al. showed that both elagolix and leuprolide were effective in reducing endometriosis-associated pain (11). Similarly, the TERRA trial conducted by D’Hooghe et al. demonstrated that ASP1707 produced dose-dependent improvements in OPP, dysmenorrhea, and NMPP (12). Consistent findings were also reported in an earlier phase II randomized controlled trial of elagolix. In that study, patients received oral elagolix at doses of 150 mg or 250 mg, and both treatment groups showed significantly greater reductions in dysmenorrhea and NMPP scores at weeks 8 and 12 compared with placebo (17). Therefore, this meta-analysis primarily reflects the relative efficacy of GnRH-a and GnRH-ant, rather than determining whether GnRH-ant provide superior analgesic efficacy.

### 4.2 Pharmacological mechanism and efficacy difference

The differences in pain-related outcomes between the two drug type are also pharmacologically plausible. GnRH-a initially stimulate pituitary secretion of LH and FSH; however, with continued administration, pituitary GnRH receptors gradually undergo desensitization and downregulation, resulting in sustained suppression of gonadotropin secretion. This subsequently leads to marked suppression of ovarian function, reductions in estrogen and progesterone levels, and the establishment of a relatively stable hypogonadotropic state (18).

By contrast, GnRH-ant act primarily by competitively binding to GnRH receptors on pituitary gonadotrophs, thereby directly blocking GnRH-mediated signaling and reducing gonadotropin secretion (19). Importantly, the magnitude of this suppression is drug- and dose-dependent. A previous pharmacodynamic study of elagolix showed that serum FSH, LH, and estradiol levels declined within hours after the first dose, with a clear dose-dependent pattern: lower doses produce partial suppression, whereas higher doses result in progressively greater hormonal suppression, approaching near-complete suppression at sufficiently high doses (20). Thus, compared with leuprolide, lower-dose GnRH-ant regimens generally allow partial preservation of estradiol production, resulting in a more moderate degree of hormonal suppression. This may reduce hypoestrogenism-related adverse effects, particularly bone loss, while maintaining sufficient symptom control. However, incomplete ovarian suppression may also explain the relatively smaller improvements observed in dysmenorrhea, menstrual blood loss, and other pain-related outcomes.

Consistent with this interpretation, D’Hooghe et al. reported that ASP1707 reduced estradiol levels into the target therapeutic range but achieved less suppression than leuprolide, accompanied by a smaller decline in BMD. Similarly, Ács et al. observed lower lumbar spine BMD loss after 12 weeks with elagolix 150 mg and 250 mg compared with leuprolide (−1.05%, −0.80%, and −1.63%, respectively). These findings are consistent with the concept that the extent of estradiol suppression may influence the balance between therapeutic efficacy and hypoestrogenic adverse effects. Previous studies have proposed an estrogen threshold hypothesis, suggesting that different tissues may respond differently to varying degrees of estradiol suppression (21). However, the specific estradiol ranges proposed in previous reports were not directly evaluated in the present meta-analysis.

From this theoretical perspective, effective treatment may not necessarily require complete suppression of estradiol to postmenopausal levels. Preserving a certain degree of estrogenic activity may still provide meaningful pain relief while reducing the bone loss associated with excessive estrogen suppression. This may constitute one of the pharmacological mechanisms by which some GnRH-ant achieve a balance between therapeutic efficacy and skeletal safety.

### 4.3 BMD findings

In contrast to pain-related outcomes, BMD outcomes consistently favored GnRH-ant. This pattern was observed in both the 12-week and 24-week subgroups, although the larger effect estimate in the 24-week subgroup should be interpreted cautiously. Differences in treatment duration may theoretically magnify the impact of estrogen suppression on bone metabolism over time; however, the regimens, doses, and study characteristics differed between subgroups, making it difficult to attribute this finding solely to treatment duration. It is worth emphasizing that the differences we observed only represent short-term changes in BMD and should not be interpreted as a reduction in the risk of long-term skeletal complications.

The BMD advantage of GnRH-ant should also not be considered uniform across all agents and regimens. Notably, higher-dose GnRH-ant regimens that produce more profound estrogen suppression may still result in clinically relevant bone loss. Phase III trials of elagolix demonstrated a clear dose- and duration-dependent effect (22). Taken together, the apparent BMD advantage of GnRH-ant over GnRH-a should remain closely dependent on the specific agent, administered dose, and duration of treatment.

None of the included trials incorporated add-back therapy in the GnRH agonist comparator arms. The included trials mainly compared GnRH-ant monotherapy with leuprolide monotherapy, which may not fully represent current clinical practice. GnRH-a are frequently combined with estrogen and/or progestogen add-back therapy to reduce hypoestrogenic symptoms and bone loss during prolonged treatment, and current ESHRE recommendations support this approach when appropriate. Therefore, the BMD differences observed in this meta-analysis primarily reflect the effects of monotherapy and should not be directly extrapolated to comparisons between GnRH-ant and contemporary GnRH-a regimens incorporating add-back therapy (3).

In recent years, a fixed-dose combination of relugolix, estradiol, and norethisterone acetate has also been increasingly introduced into clinical practice. Two phase III, multicenter, randomized, double-blind, placebo-controlled trials enrolled a total of 1,261 premenopausal women aged 18–50 years with moderate-to-severe endometriosis-associated pain. After 24 weeks of treatment, the dysmenorrhea response rate in the relugolix combination therapy group was 75% in both trials, compared with 27% and 30% in the respective placebo groups. The corresponding response rates for NMPP were 58% and 66% with combination therapy, compared with 40% and 43% with placebo, with statistically significant between-group differences (23).

These developments indicate that the clinical use of GnRH-ant is no longer limited to monotherapy but increasingly emphasizes preserving therapeutic efficacy while minimizing adverse effects related to hypoestrogenism. Accordingly, the BMD differences observed in this meta-analysis are primarily applicable to comparisons between GnRH-ant monotherapy and leuprolide monotherapy and should not be directly extrapolated to contemporary add-back regimens using the fixed combination of relugolix, estradiol, and norethisterone acetate. Further studies directly comparing GnRH-ant with contemporary GnRH-a plus add-back strategies are warranted to clarify their relative long-term skeletal effects.

### 4.4 Menstrual outcomes, analgesic use, and quality of life

In terms of analgesic use, GnRH-a can reduce the proportion of days on which patients use analgesics. Although the magnitude of the between-group difference was relatively small, the overall findings were generally consistent and aligned with the change observed for the pain-related outcomes described above. However, the percentage of days of analgesic use cannot be considered fully equivalent to pain severity. The included trials may have differed in the types of rescue medications permitted and the criteria governing their use. In addition, patients’ individual medication-taking behaviors and regional differences in clinical prescribing practices may also have influenced this outcome. Therefore, a reduction in analgesic use may serve as supportive evidence of pain improvement, but it should not be interpreted in isolation as a definitive indicator of comparative efficacy between the two drug classes.

Menstrual blood loss outcomes also favored GnRH-a, which may be explained by stronger ovarian suppression and a greater likelihood of reducing endometrial activity or inducing amenorrhea. Nevertheless, substantial heterogeneity was observed, and most available data were derived from relugolix studies. Leave-one-out sensitivity analysis indicated that the pooled estimates were not substantially altered after excluding individual studies, suggesting that the findings were not predominantly driven by any single trial. However, the result showed fluctuations in effect size, warranting cautious interpretation. Thus, the superiority of GnRH-a for menstrual bleeding control remains uncertain and requires further confirmation.

Quality-of-life outcomes showed domain-specific differences. GnRH-a demonstrated modest benefits in the Pain and Control and Powerlessness domains, while no significant differences were observed in Emotional Wellbeing, Social Support, or Self-image. These findings may reflect the different trajectories of symptom relief and psychosocial recovery. Pain improvement may occur relatively quickly after treatment initiation, whereas psychological and social aspects of quality of life are influenced by multiple factors, including disease duration, infertility concerns, interpersonal relationships, baseline psychological status, and social support. Therefore, short-term hormonal suppression and pain relief alone may not be sufficient to produce broad improvements in psychosocial functioning.

### 4.5 Clinical implications

Taken together, the findings of the present study do not provide sufficient evidence to support either GnRH-a or GnRH-ant as the universally preferred treatment for all patients. For patients with more severe pain, prominent dysmenorrhea, and a need for relatively profound ovarian suppression over the short term, particularly those at low risk of bone loss, GnRH-a may offer some advantages in symptom control. In contrast, GnRH-ant may be more appropriate for patients who prefer oral administration, wish to avoid the initial hormonal flare associated with GnRH-a, or require greater flexibility in adjusting the degree of ovarian suppression according to therapeutic response and tolerability. This option may be particularly relevant for patients at increased risk of bone loss. Harada et al. also observed that menstruation resumed earlier after discontinuation of relugolix than that of leuprolide (14). This finding may have practical clinical relevance for patients who desire a more rapid recovery of menstrual function after treatment cessation. However, earlier return of menstruation should not be interpreted as evidence of improved fertility or reproductive outcomes, as these endpoints were not directly assessed.

At present, the ESHRE guideline considers both GnRH-a and GnRH-ant as second-line treatment options, generally for patients with an inadequate response to combined hormonal contraceptives or progestogens. The guideline also emphasizes that treatment selection should take into account patient preferences, adverse effects, previous individual treatment response, treatment costs, and drug availability (3). Accordingly, clinical decision-making should incorporate multiple patient-specific factors, including the type and severity of pain, response to previous therapies, baseline BMD, age, fertility plans, preferences regarding route of administration, anticipated adherence to long-term treatment, and whether the patient is an appropriate candidate for add-back therapy.

### 4.6 Strengths and limitations

This study has several strengths. The analysis was restricted to randomized controlled trials with leuprolide-based GnRH-a, minimizing uncertainty inherent to indirect comparisons. Additionally, the evaluation extended beyond pain outcomes to encompass physical signs, analgesic use, menstrual bleeding, BMD and disease-specific quality of life, enabling a comprehensive assessment of the efficacy and safety profiles of GnRH-ant and leuprolide-based GnRH-a.

Several limitations warrant consideration. First, the number of eligible trials was modest, and many were phase II dose-finding studies with follow-up largely confined to 12–24 weeks; consequently, current evidence primarily supports short-term comparisons, whereas long-term outcomes—post-discontinuation symptom recurrence, BMD recovery, fracture risk, treatment persistence, and reproductive outcomes—remain uncertain. Second, subgroup analysis should be interpreted cautiously. Although analysis based on treatment duration and GnRH-ant type were prespecified according to clinical considerations, the possibility of type I error due to multiple comparisons cannot be excluded. Third, Substantial inter-study variability in assessment tools, outcome definitions, GnRH-ant doses, and treatment regimens likely contributed to observed heterogeneity. Fourth, all GnRH-a comparators were leuprolide, restricting our findings should be interpreted primarily as comparisons between GnRH-ant and leuprolide-based GnRH-a therapy, and their applicability to other GnRH-a such as goserelin or triptorelin remains uncertain. Caution similarly applies to GnRH-ant: some agents (e.g., ASP1707) are not widely used clinically, and evaluated doses of relugolix and elagolix may not fully correspond to currently approved regimens. Fifth, although several results showed statistically significant differences, statistical significance alone does not necessarily represent clinically meaningful superiority. For some pain-related outcomes, including OPP and dyspareunia, the magnitude of the pooled effect is relatively small. Future studies that incorporate validated clinical thresholds (such as the minimal clinically important difference) are needed to clarify the practical significance of these observed differences. Sixth, although publication bias and small-study effects may influence the results of the meta-analysis, we did not conduct a formal test for publication bias because only six randomized controlled trials were included. Therefore, the possibility of publication bias or small-study effects cannot be ruled out, and the pooled estimates should be interpreted with caution. Seventh, although safety outcomes were evaluated descriptively, heterogeneity in adverse event reporting and insufficient data availability precluded pooled analysis of individual safety endpoints. Finally, although pooling GnRH-ant as a single class enhances statistical comparability, individual agents differ in pharmacokinetic properties, dosing regimens, and the extent of ovarian suppression. Therefore, the pooled effects should not be interpreted as indicating identical efficacy or safety profiles across all GnRH-ants.

### 4.7 Future research directions

Future research should prioritize long-term, head-to-head RCTs employing contemporary dosing and standardized add-back regimens. Harmonized definitions and assessment tools are required for pain, analgesic use, menstrual outcomes, and quality of life. Beyond continuous measures, reporting the proportion of patients achieving minimal clinically important improvement would enhance clinical interpretability of treatment effects. Trials should also extend follow-up beyond the treatment period to capture symptom recurrence, menstrual and ovulatory recovery, BMD trajectories, adherence, adverse events, and reproductive outcomes.

Based on current direct comparative evidence, GnRH-a offers modest advantages for dysmenorrhea and select pain-related outcomes, whereas GnRH-ant is associated with less BMD decline. Most between-group differences were small and their clinical relevance remains uncertain. Given the limited trial count, substantial heterogeneity, and short follow-up, current evidence does not support overall superiority of either class. Rather, GnRH-a and GnRH-ant represent options with distinct benefit–risk profiles, and selection should be individualized based on pain severity, skeletal health, reproductive goals, treatment preference, and add-back therapy feasibility.

## 5 Conclusion

This systematic review and meta-analysis of RCTs comparing GnRH-ant and leuprolide-based GnRH-a indicates that neither agent is universally superior; rather, they possess distinct strengths. GnRH-a, mainly leuprolide, appeared to provide better relief of several endometriosis-related pain outcomes, particularly dysmenorrhea, whereas GnRH-ant was associated with less BMD decline. Most between-group differences were small-to-moderate, however, and substantial heterogeneity in some outcomes suggests that statistical significance does not invariably translate into clinically meaningful benefit. Comparative effects may further depend on treatment duration, GnRH-ant subtype, and dosing regimens. Because all included trials employed leuprolide as the GnRH-a comparator and most assessed monotherapy, these findings warrant cautious interpretation and should not be generalized to other GnRH-a regimens or add-back-inclusive strategies. In clinical practice, selection between GnRH-a and GnRH-ant should be individualized based on pain severity, baseline bone health, reproductive plans, treatment preference, and adverse-effect tolerance. leuprolide-based GnRH-a may be preferred for patients requiring short-term pain suppression, while GnRH-ant is appropriate for those concerned about bone loss, favoring oral administration, or needing to avoid hormonal flare. But these findings represent class-level effects, and differences among individual antagonists require further investigation. Future head-to-head trials with standardized outcomes, extended follow-up, and contemporary add-back strategies are needed to identify patients most likely to benefit from specific agents and regimens.

## Data Availability

The original contributions presented in the study are included in the article/**Supplementary Material**. Further inquiries can be directed to the corresponding author.
