# Supplementary material for: Gonadotropin-releasing hormone antagonists versus agonists for endometriosis: a systematic review and meta-analysis of randomized controlled trials
Source: Front Endocrinol (Lausanne). 2026 Oct 14;17:1979544. doi: 10.3389/fendo.2026.1979544 (PMC13714813; doi:10.3389/fendo.2026.1979544)
Supplement: Supplementary file 1 [file SupplementaryFile1.docx]

Supplementary Material

# 1 Supplementary Data

## 1.1 Search strategy for PubMed

The search strategy was developed using a combination of Medical Subject Headings (MeSH) terms and free-text keywords related to endometriosis, gonadotropin-releasing hormone agonists (GnRH-a), gonadotropin-releasing hormone antagonists (GnRH-ant), and randomized controlled trials. The detailed search strategy was as follows: #1 to 3 are for retrieving endometriosis, #4 to 11 are for retrieving GnRH-ant, #12 to 20 are for retrieving GnRH-a, #21 to 24 are for retrieving document types, #25 is for retrieving animal experiments, and #26 to 27 are combinations of the above retrieval results:

#1 "Endometriosis"[Mesh]

#2 "Endometrioses"[Title/Abstract] OR "Endometrioma"[Title/Abstract] OR "Endometriomas"[Title/Abstract].

#3 #1 OR #2

#4 "Gonadotropin-Releasing Hormone/antagonists and inhibitors"[Mesh]

#5 ("GnRH antagonist*"[Title/Abstract]) OR ("gonadotropin-releasing hormone antagonist*"[Title/Abstract])

#6 ("Elagolix"[Mesh]) OR ("R-(+)-4-(2-(5-(2-fluoro-3-methoxyphenyl)-3-(2-fluoro-6-(trifluoromethyl)benzyl)-4-methyl-2,6-dioxo-3,6-dihydro-2H-pyrimidin-1-yl)-1-phenylethylamino)butyrate "[Title/Abstract]) OR ("Orilissa"[Title/Abstract]) OR ("elagolix sodium"[Title/Abstract])

#7 ("Relugolix"[Mesh]) OR ("TAK 385"[Title/Abstract]) OR ("TAK-385"[Title/Abstract]) OR ("TAK385"[Title/Abstract]) OR ("1-(4-(1-(2,6-difluorobenzyl)-5-((dimethylamino)methyl)-3-(6-methoxypyridazin-3-yl)-2,4-dioxo-1,2,3,4-tetrahydrothieno(2,3-d)pyrimidin-6-yl)phenyl)-3-methoxyurea"[Title/Abstract]) OR ("orgovyx"[Title/Abstract])

#8 ("Linzagolix"[Mesh]) OR ("3-(5-((2,3-difluoro-6-methoxyphenyl)methoxy)-2-fluoro-4-methoxyphenyl)-2,4-dioxo-1H-thieno(3,4-d)pyrimidine-5-carboxylic acid"[Title/Abstract]) OR ("OBE-2109"[Title/Abstract]) OR ("OBE2109"[Title/Abstract]) OR ("KLH2109"[Title/Abstract]) OR ("KLH-2109"[Title/Abstract])

#9 ("Cetrorelix"[Mesh]) OR ("LHRH, N-Ac-1-Nal(2)-2-Phe(pCl)-3-Pal(3)-6-Cit-10-Ala-"[Title/Abstract]) OR ("LHRH, N-acetyl-1-(3-(2-naphthyl)alanyl)-2-(4-chlorophenylalanyl)-3-(3-(3-pyridyl)alanyl)-6-citrulline-10-alanine-"[Title/Abstract]) OR ("N-acetyl-1-(3-(2-naphthyl)alanine)-2-(4-chlorophenylalanine)-3-(3-(3-pyridyl)alanine)-6-citrulline-10-alanine-LHRH"[Title/Abstract]) OR ("SB 75"[Title/Abstract]) OR ("SB-75"[Title/Abstract]) OR ("Cetrotide"[Title/Abstract]) OR ("cetrorelix acetate"[Title/Abstract]) OR ("cetrorelix pamoate"[Title/Abstract])

#10 ("Ganirelix"[Mesh]) OR ("GnRH, N-Ac-2-Nal(1)-4-Cl-Phe(2)-3-Pal(3)-Et2-hArg(6,8)-AlaNH2(10)-"[Title/Abstract]) OR ("LHRH, N-acetyl-2-naphthylalanyl(1)-(4-chlorophenylalanyl)(2)-3-pyrdinylalanyl(3)-diethylhomoarginyl(6,8)-alaninamide(10)-"[Title/Abstract]) OR ("LHRH,N-Ac-2-Nal(1)-4-Cl-Phe(2)-3-Pal(3)-Et2-hArg(6,8)-AlaNH2(10)-"[Title/Abstract]) OR ("N-Ac-(2-naphthyl)Ala-2-(4-Cl-Phe)-3-(3-pyridinyl-Ala)-6,8-Et2-hArg-10-AlaNH2-LHRH"[Title/Abstract]) OR ("RS 26306"[Title/Abstract]) OR ("RS-26306"[Title/Abstract]) OR ("Antagon"[Title/Abstract]) OR ("orgalutran"[Title/Abstract]) OR ("ganirelix acetate"[Title/Abstract]) OR ("N-acetyl-3-(2-naphthyl)-D-alanyl-p-chloro-D-phenylalanyl-3-(3-pyridyl)-D-alanyl-L-seryl-L-tyrosyl-N(sup 6)-(N,N'-diethylamidino)-D-lysyl-L-leucyl-N(sup 6)-(N,N'-diethylamidino)-L-lysyl-L-prolyl-D-alaninamide diacetate (salt)"[Title/Abstract]) OR ("ganirelix diacetate"[Title/Abstract]) OR ("RS-26306-298"[Title/Abstract])

#11 #4 OR #5 OR #6 OR #7 OR #8 OR #9 OR #10

#12 "Gonadotropin-Releasing Hormone/agonists"[Mesh]

#13 ("GnRH agonist*"[Title/Abstract]) OR ("gonadotropin-releasing hormone agonist*"[Title/Abstract])

#14 ("Leuprolide"[Mesh]) OR ("Leuprorelin"[Title/Abstract]) OR ("TAP-144"[Title/Abstract]) OR ("TAP144"[Title/Abstract]) OR ("TAP 144"[Title/Abstract]) OR ("Enantone"[Title/Abstract]) OR ("Leuprolide, (L-Leu)-Isomer"[Title/Abstract]) OR ("Leuprolide, (DL-Leu)-Isomer"[Title/Abstract]) OR ("Leuprolide Monoacetate"[Title/Abstract]) OR ("Monoacetate, Leuprolide"[Title/Abstract]) OR ("Leuprolide Acetate"[Title/Abstract]) OR ("Acetate, Leuprolide"[Title/Abstract]) OR ("Lupron"[Title/Abstract]) OR ("A-43818"[Title/Abstract]) OR ("A43818"[Title/Abstract]) OR ("A 43818"[Title/Abstract])

#15 ("Goserelin"[Mesh]) OR ("Zoladex"[Title/Abstract]) OR ("ICI-118630"[Title/Abstract]) OR ("ICI118630"[Title/Abstract]) OR ("ICI 118630"[Title/Abstract]) OR ("Goserelin Acetate"[Title/Abstract]) OR ("Acetate, Goserelin"[Title/Abstract])

#16 ("Triptorelin Pamoate"[Mesh]) OR ("Pamoate, Triptorelin"[Title/Abstract]) OR ("Decapeptyl"[Title/Abstract]) OR ("Decapeptyl Depot"[Title/Abstract]) OR ("Decapeptyl Trimestral"[Title/Abstract]) OR ("Trimestral, Decapeptyl"[Title/Abstract]) OR ("Decapeptyl LP"[Title/Abstract]) OR ("AY-25650"[Title/Abstract]) OR ("AY25650"[Title/Abstract]) OR ("AY 25650"[Title/Abstract]) OR ("CL-118532"[Title/Abstract]) OR ("CL118532"[Title/Abstract]) OR ("CL 118532"[Title/Abstract]) OR ("Wy-42462"[Title/Abstract]) OR ("Wy42462"[Title/Abstract]) OR ("Wy 42462"[Title/Abstract]) OR ("Trelstar"[Title/Abstract]) OR ("Triptorelin"[Title/Abstract]) OR ("D-Trp-6-LH-RH"[Title/Abstract]) OR ("6-D-Tryptophan-Luteinizing Hormone-Releasing Factor (Pig)"[Title/Abstract]) OR ("GnRH, Trp(6)-"[Title/Abstract]) OR ("LHRH, Tryptophyl(6)-"[Title/Abstract]) OR ("LHRH, Trp(6)-"[Title/Abstract]) OR ("Triptorelin Embonate"[Title/Abstract]) OR ("Embonate, Triptorelin"[Title/Abstract])

#17 ("Nafarelin"[Mesh]) OR ("Nafarelin Monoacetate"[Title/Abstract]) OR ("Nafarelin Acetate"[Title/Abstract]) OR ("Nafarelin Acetate, Hydrate"[Title/Abstract]) OR ("Synarel"[Title/Abstract]) OR ("RS-94991-298"[Title/Abstract]) OR ("RS94991298"[Title/Abstract]) OR ("RS 94991 298"[Title/Abstract])

#18 ("Buserelin"[Mesh]) OR ("Receptal"[Title/Abstract]) OR ("Tiloryth"[Title/Abstract]) OR ("Buserelin Acetate"[Title/Abstract]) OR ("Acetate, Buserelin"[Title/Abstract]) OR ("HOE-766"[Title/Abstract]) OR ("HOE766"[Title/Abstract]) OR ("HOE 766"[Title/Abstract]) OR ("Suprecur"[Title/Abstract]) OR ("Suprefact"[Title/Abstract]) OR ("Profact"[Title/Abstract]) OR ("Bigonist"[Title/Abstract])

#19 #12 OR #13 OR #14 OR #15 OR #16 OR #17 OR #18

#20 #11 OR #19

#21 "Randomized Controlled Trial" [Publication Type]

#22 "Controlled Clinical Trial" [Publication Type]

#23 ("randomized"[Title/Abstract] OR ("randomised"[Title/Abstract]) OR ("placebo"[Title/Abstract]) OR ("randomly"[Title/Abstract]) OR ("trial"[Title/Abstract]) OR ("clinical trial"[Title/Abstract]))

#24 #21 OR #22 OR #23

#25 ("animals"[Mesh]) NOT ("humans"[Mesh])

#26 #3 AND #20 AND #24

#27 final search strategy: #26 NOT #25

## 1.2 Search strategy for Web of Science:

The search strategy in Web of Science combines terms related to endometriosis, GnRH-ant, GnRH-a and randomized controlled trials, and uses topic search (TS) to identify relevant literature records. The specific search strategy is as follows: #1 refers to the search for endometriosis, #2 refers to the search for GnRH-ant, #3 refers to the search for GnRH-a, #4 refers to the search for literature types, and #5 is the combination of the above search results:

#1 TS= (endometriosis OR "endometriotic lesion*" OR "endometrioma*" OR "endometriosis pain" OR "pelvic endometriosis")

#2 TS=("GnRH antagonist*" OR "gonadotropin releasing hormone antagonist*" OR "GnRH antag*" OR "antagonist* of gonadotropin releasing hormone" OR "Elagolix" OR "Relugolix" OR "Linzagolix" OR "Cetrorelix" OR "Ganirelix")

#3 TS=("GnRH agonist*" OR "gonadotropin releasing hormone agonist*" OR "GnRH ago*" OR "agonist* of gonadotropin releasing hormone" OR "Leuprolide" OR "Leuprorelin" OR "Goserelin" OR "Triptorelin" OR "Nafarelin" OR "Buserelin")

#4 TS=("randomized controlled trial" OR "randomised controlled trial" OR RCT OR "randomized trial" OR "randomised trial" OR "clinical trial")

#5 final search strategy: #1 AND #2 AND #3 AND #4

## 1.3 Search strategy for Embase:

The search strategy in the Embase database was developed by combining the controlled vocabulary (Emtree terms) with free-text terms related to the four core concepts of endometriosis, GnRH-ant, GnRH-a, and randomized controlled trials. Emtree terms were expanded for search where appropriate, and synonyms under each concept were combined using the Boolean operator "OR". The four concept modules were then combined using "AND". The specific search strategy is as follows: #1 to 7 are for searching endometriosis, #8 to 14 are for searching GnRH-ant, #15 to 23 are for searching GnRH-a, #24 to 29 are for searching literature types, and #30 is the combination of the above search results:

#1 'endometriosis'/exp

#2 endometriosis:ti,ab,kw

#3 endometriotic lesion*:ti,ab,kw

#4 endometrioma*:ti,ab,kw

#5 'pelvic endometriosis':ti,ab,kw

#6 'endometriosis pain':ti,ab,kw

#7 #1 OR #2 OR #3 OR #4 OR #5 OR #6

#8 'gonadotropin releasing hormone antagonist'/exp

#9 ('gonadotropin releasing hormone antagonist*'):ti,ab,kw

#10 GnRH antag*:ti,ab,kw

#11 antagonist* of gonadotropin releasing hormone:ti,ab,kw

#12 elagolix:ti,ab,kw

#13 relugolix:ti,ab,kw

#14 #8 OR #9 OR #10 OR #11 OR #12 OR #13

#15 'gonadotropin releasing hormone agonist'/exp

#16 ('gonadotropin releasing hormone agonist*'):ti,ab,kw

#17 GnRH ago*:ti,ab,kw

#18 agonist* of gonadotropin releasing hormone:ti,ab,kw

#19 leuprolide:ti,ab,kw

#20 goserelin:ti,ab,kw

#21 triptorelin:ti,ab,kw

#22 nafarelin:ti,ab,kw

#23 #15 OR #16 OR #17 OR #18 OR #19 OR #20 OR #21 OR #22

#24 'randomized controlled trial'/exp

#25 randomized controlled trial:ti,ab,kw

#26 random*:ti,ab,kw

#27 placebo:ti,ab,kw

#28 clinical trial:ti,ab,kw

#29 #24 OR #25 OR #26 OR #27 OR #28

#30 final search strategy: #7 AND (#14 AND #23) AND #29

## 1.4 Search strategy for Cochrane Library:

The search strategy in the Cochrane Library database consists of four concept groups: (1) terms related to endometriosis, (2) terms related to GnRH-ant, (3) terms related to GnRH-a, (4) terms related to randomized controlled trials. Synonymous terms within each concept group are combined using the Boolean operator "OR", and the four concept groups are combined using "AND". The specific search strategy is as follows: #1 refers to the search for endometriosis, #2 refers to the search for GnRH-ant, #3 refers to the search for GnRH-a, #4 refers to the search for literature types, and #5 refers to the combination of the above search results:

#1 (endometriosis OR endometriotic OR endometrioma OR "pelvic endometriosis" OR "endometriosis pain")

#2 ((GnRH antagonist*) OR (Gonadotropin-releasing hormone antagonist*) OR Elagolix OR Relugolix OR Linzagolix OR Cetrorelix OR Ganirelix)

#3 ((GnRH agonist*) OR (Gonadotropin-releasing hormone agonist*) OR Leuprorelin OR Leuprolide OR Goserelin OR Triptorelin OR Nafarelin OR Buserelin)

#4 (randomized OR randomised OR placebo OR "randomized controlled trial" OR "randomised controlled trial" OR RCT)

#5 final search strategy: #1 AND #2 AND #3 AND #4

# 2 Supplementary Figures and Tables

## 2.1 Supplementary Figures

**Supplementary Figure 1.** Leave-one-out sensitivity analysis for dysmenorrhea.

**Supplementary Figure 2.** Leave-one-out sensitivity analysis for nonmenstrual pelvic pain (NMPP).

**Supplementary Figure 3.** Leave-one-out sensitivity analysis for overall pelvic pain (OPP).

**Supplementary Figure 4.** Leave-one-out sensitivity analysis for dyspareunia.

**Supplementary Figure 5.** Leave-one-out sensitivity analysis for pelvic tenderness.

**Supplementary Figure 6.** Leave-one-out sensitivity analysis for bone mineral density (BMD).

**Supplementary Figure 7.** Forest plot of changes from baseline in scores for induration of the Douglas cavity, stratified by treatment duration.

**Supplementary Figure 8.** Forest plot of changes from baseline in scores for induration of the Douglas cavity, stratified by type of GnRH-ant.

**Supplementary Figure 9.** Forest plot of changes from baseline in peak score for endometriosis pain, stratified by treatment duration.

**Supplementary Figure 10.** Forest plot of changes from baseline in peak score for endometriosis pain, stratified by type of GnRH-ant.

**Supplementary Figure 11.** Forest plot of changes from baseline in the percentage of days of analgesic use, stratified by treatment duration.

**Supplementary Figure 12.** Forest plot of changes from baseline in the percentage of days of analgesic use, stratified by type of GnRH-ant.

**Supplementary Figure 13.** Forest plot of changes from baseline in menstrual blood loss, stratified by treatment duration.

**Supplementary Figure 14.** Forest plot of changes from baseline in menstrual blood loss, stratified by type of GnRH-ant.

**Supplementary Figure 15.** Leave-one-out sensitivity analysis for menstrual blood loss.

**Supplementary Figure 16.** Forest plot of changes from baseline in the Pain domain, stratified by treatment duration.

**Supplementary Figure 17.** Forest plot of changes from baseline in the Control and Powerlessness domain, stratified by treatment duration.

**Supplementary Figure 18.** Forest plot of changes from baseline in the Emotional Wellbeing domain, stratified by treatment duration.

**Supplementary Figure 19.** Forest plot of changes from baseline in the Social Support domain, stratified by treatment duration.

**Supplementary Figure 20.** Forest plot of changes from baseline in the Self-image domain, stratified by treatment duration.

**Supplementary Figure 21.** Forest plot of changes from baseline in the Pain domain, stratified by type of GnRH-ant.

**Supplementary Figure 22.** Forest plot of changes from baseline in the Control and Powerlessness domain, stratified by type of GnRH-ant.

**Supplementary Figure 23.** Forest plot of changes from baseline in the Emotional Wellbeing domain, stratified by type of GnRH-ant.

**Supplementary Figure 24.** Forest plot of changes from baseline in the Social Support domain, stratified by type of GnRH-ant.

**Supplementary Figure 25.** Forest plot of changes from baseline in the Self-image domain, stratified by type of GnRH-ant.

**Supplementary Figure 26.** Chemical structures of GnRH antagonists and GnRH agonists included in this review. Where A represents leuprorelin (PubChem CID: 657181), B represents elagolix (PubChem CID: 11250647), C represents relugolix (PubChem CID: 10348973), and D represents ASP1707 (PubChem CID: 135565248) (S1).

## 2.2 Supplementary Tables

Abbreviations: T indicates intervention group; C indicates control group; SD represents standard deviation.

| **Study** | **T sample** | **T mean** | **T sd** | **C sample** | **C mean** | **C sd** |
| --- | --- | --- | --- | --- | --- | --- |
| Acs N, 2015 (12weeks, Elagolix 150mg) | 42 | -0.76 | 0.5185 | 44 | -1.14 | 0.5307 |
| Acs N, 2015 (12weeks, Elagolix 250mg) | 42 | -0.87 | 0.5185 | 44 | -1.14 | 0.5307 |
| D'Hooghe T, 2019 (12weeks, ASP1707 3mg) | 86 | -1.12 | 0.9463 | 89 | -2.08 | 0.8423 |
| D'Hooghe T, 2019 (12weeks, ASP1707 5mg) | 91 | -1.20 | 0.9004 | 89 | -2.08 | 0.8423 |
| D'Hooghe T, 2019 (12weeks, ASP1707 10mg) | 90 | -1.53 | 0.9438 | 89 | -2.08 | 0.8423 |
| D'Hooghe T, 2019 (12weeks, ASP1707 15mg) | 88 | -1.70 | 0.9094 | 89 | -2.08 | 0.8423 |
| Osuga Y, 2020 (12weeks, Relugolix 10mg) | 103 | -0.48 | 0.6500 | 80 | -1.17 | 0.4700 |
| Osuga Y, 2020 (12weeks, Relugolix 20mg) | 100 | -0.76 | 0.6700 | 80 | -1.17 | 0.4700 |
| Osuga Y, 2020 (12weeks, Relugolix 40mg) | 103 | -1.16 | 0.4900 | 80 | -1.17 | 0.4700 |
| Harada T, 2021 (24weeks, Relugolix 40mg) | 164 | -2.10 | 0.0000 | 155 | -2.10 | 0.0000 |
| Osuga Y, 2021 (24weeks, Relugolix 10mg) | 103 | -0.50 | 0.6700 | 80 | -1.20 | 0.4700 |
| Osuga Y, 2021 (24weeks, Relugolix 20mg) | 100 | -0.80 | 0.6500 | 80 | -1.20 | 0.4700 |
| Osuga Y, 2021 (24weeks, Relugolix 40mg) | 103 | -1.10 | 0.5000 | 80 | -1.20 | 0.4700 |

**Supplementary Table 1.** Extracted data for dysmenorrhea.

| **Study** | **T sample** | **T mean** | **T sd** | **C sample** | **C mean** | **C sd** |
| --- | --- | --- | --- | --- | --- | --- |
| Acs N, 2015 (12weeks, Elagolix 150mg) | 42 | -0.35 | 0.3888 | 44 | -0.55 | 0.3980 |
| Acs N, 2015 (12weeks, Elagolix 250mg) | 42 | -0.34 | 0.3888 | 44 | -0.55 | 0.3980 |
| D'Hooghe T, 2019 (12weeks, ASP1707 3mg) | 86 | -0.81 | 0.7807 | 89 | -1.26 | 0.7701 |
| D'Hooghe T, 2019 (12weeks, ASP1707 5mg) | 91 | -0.98 | 0.7544 | 89 | -1.26 | 0.7701 |
| D'Hooghe T, 2019 (12weeks, ASP1707 10mg) | 90 | -1.01 | 0.7744 | 89 | -1.26 | 0.7701 |
| D'Hooghe T, 2019 (12weeks, ASP1707 15mg) | 88 | -1.09 | 0.7419 | 89 | -1.26 | 0.7701 |
| Harada T, 2021 (24weeks, Relugolix 40mg) | 164 | -1.40 | 1.2806 | 155 | -1.40 | 1.2450 |

**Supplementary Table 2.** Extracted data for NMPP.

| **Study** | **T sample** | **T mean** | **T sd** | **C sample** | **C mean** | **C sd** |
| --- | --- | --- | --- | --- | --- | --- |
| Acs N, 2015 (12weeks, Elagolix 150mg) | 42 | -1.34 | 1.3610 | 44 | -1.81 | 1.3266 |
| Acs N, 2015 (12weeks, Elagolix 250mg) | 42 | -1.47 | 1.3610 | 44 | -1.81 | 1.3266 |
| Harada T, 2021 (24weeks, Relugolix 40mg) | 171 | -11.80 | 8.6306 | 164 | -13.42 | 8.7082 |
| Osuga Y, 2021 (24weeks, Relugolix 10mg) | 84 | -6.80 | 10.5600 | 69 | -12.70 | 12.5700 |
| Osuga Y, 2021 (24weeks, Relugolix 20mg) | 78 | -9.00 | 11.8400 | 69 | -12.70 | 12.5700 |
| Osuga Y, 2021 (24weeks, Relugolix 40mg) | 89 | -11.90 | 11.2600 | 69 | -12.70 | 12.5700 |

**Supplementary Table 3.** Extracted data for OPP.

| **Study** | **T sample** | **T mean** | **T sd** | **C sample** | **C mean** | **C sd** |
| --- | --- | --- | --- | --- | --- | --- |
| D'Hooghe T, 2019 (12weeks, ASP1707 3mg) | 86 | -0.68 | 1.0409 | 89 | -0.92 | 1.0589 |
| D'Hooghe T, 2019 (12weeks, ASP1707 5mg) | 91 | -0.89 | 1.0221 | 89 | -0.92 | 1.0589 |
| D'Hooghe T, 2019 (12weeks, ASP1707 10mg) | 90 | -0.79 | 1.0165 | 89 | -0.92 | 1.0589 |
| D'Hooghe T, 2019 (12weeks, ASP1707 15mg) | 88 | -0.98 | 1.0290 | 89 | -0.92 | 1.0589 |
| Osuga Y, 2020 (12weeks, Relugolix 10mg) | 41 | -0.08 | 0.4900 | 20 | -0.36 | 0.5200 |
| Osuga Y, 2020 (12weeks, Relugolix 20mg) | 38 | -0.10 | 0.5700 | 20 | -0.36 | 0.5200 |
| Osuga Y, 2020 (12weeks, Relugolix 40mg) | 34 | -0.07 | 0.4900 | 20 | -0.36 | 0.5200 |
| Harada T, 2021 (24weeks, Relugolix 40mg) | 41 | -0.40 | 0.6403 | 28 | -0.50 | 0.5292 |
| Osuga Y, 2021 (24weeks, Relugolix 10mg) | 40 | -0.20 | 0.4700 | 18 | -0.20 | 0.5600 |
| Osuga Y, 2021 (24weeks, Relugolix 20mg) | 34 | -0.20 | 0.4900 | 18 | -0.20 | 0.5600 |
| Osuga Y, 2021 (24weeks, Relugolix 40mg) | 31 | -0.20 | 0.4300 | 18 | -0.20 | 0.5600 |

**Supplementary Table 4.** Extracted data for dyspareunia.

| **Study** | **T sample** | **T mean** | **T sd** | **C sample** | **C mean** | **C sd** |
| --- | --- | --- | --- | --- | --- | --- |
| D'Hooghe T, 2019 (12weeks, ASP1707 3mg) | 86 | -0.61 | 0.7334 | 89 | -1.03 | 0.7220 |
| D'Hooghe T, 2019 (12weeks, ASP1707 5mg) | 91 | -0.80 | 0.7057 | 89 | -1.03 | 0.7220 |
| D'Hooghe T, 2019 (12weeks, ASP1707 10mg) | 90 | -0.77 | 0.7260 | 89 | -1.03 | 0.7220 |
| D'Hooghe T, 2019 (12weeks, ASP1707 15mg) | 88 | -1.03 | 0.7179 | 89 | -1.03 | 0.7220 |
| Osuga Y, 2020 (12weeks, Relugolix 10mg) | 101 | -0.50 | 0.7000 | 75 | -0.80 | 0.8000 |
| Osuga Y, 2020 (12weeks, Relugolix 20mg) | 92 | -0.70 | 0.8000 | 75 | -0.80 | 0.8000 |
| Osuga Y, 2020 (12weeks, Relugolix 40mg) | 101 | -0.90 | 0.9000 | 75 | -0.80 | 0.8000 |
| Harada T, 2021 (24weeks, Relugolix 40mg) | 162 | -0.90 | 0.9000 | 156 | -0.80 | 0.8000 |
| Osuga Y, 2021 (24weeks, Relugolix 10mg) | 79 | -0.70 | 0.8300 | 61 | -1.10 | 0.7800 |
| Osuga Y, 2021 (24weeks, Relugolix 20mg) | 74 | -0.80 | 0.7900 | 61 | -1.10 | 0.7800 |
| Osuga Y, 2021 (24weeks, Relugolix 40mg) | 87 | -1.00 | 0.9200 | 61 | -1.10 | 0.7800 |

**Supplementary Table 5.** Extracted data for pelvic tenderness.

| **Study** | **T sample** | **T mean** | **T sd** | **C sample** | **C mean** | **C sd** |
| --- | --- | --- | --- | --- | --- | --- |
| Acs N, 2015 (12weeks, Elagolix 150mg) | 41 | -1.05 | 1.9850 | 44 | -1.63 | 2.1130 |
| Acs N, 2015 (12weeks, Elagolix 250mg) | 42 | -0.80 | 2.3520 | 44 | -1.63 | 2.1130 |
| Osuga Y, 2020 (12weeks, Relugolix 10mg) | 103 | -1.00 | 1.8800 | 80 | -2.20 | 1.6700 |
| Osuga Y, 2020 (12weeks, Relugolix 20mg) | 100 | -1.30 | 2.0900 | 80 | -2.20 | 1.6700 |
| Osuga Y, 2020 (12weeks, Relugolix 40mg) | 103 | -2.10 | 2.2200 | 80 | -2.20 | 1.6700 |
| D'Hooghe T, 2019 (24weeks, ASP1707 3mg) | 67 | -1.20 | 2.3983 | 65 | -3.90 | 2.4214 |
| D'Hooghe T, 2019 (24weeks, ASP1707 5mg) | 76 | -1.20 | 2.5382 | 65 | -3.90 | 2.4214 |
| D'Hooghe T, 2019 (24weeks, ASP1707 10mg) | 65 | -1.30 | 2.3811 | 65 | -3.90 | 2.4214 |
| D'Hooghe T, 2019 (24weeks, ASP1707 15mg) | 65 | -2.30 | 2.4214 | 65 | -3.90 | 2.4214 |

**Supplementary Table 6.** Extracted data for BMD.

| **Study** | **T sample** | **T mean** | **T sd** | **C sample** | **C mean** | **C sd** |
| --- | --- | --- | --- | --- | --- | --- |
| D'Hooghe T, 2019 (12weeks, ASP1707 3mg) | 86 | -0.58 | 0.7807 | 89 | -0.85 | 0.7701 |
| D'Hooghe T, 2019 (12weeks, ASP1707 5mg) | 91 | -0.70 | 0.7787 | 89 | -0.85 | 0.7701 |
| D'Hooghe T, 2019 (12weeks, ASP1707 10mg) | 90 | -0.69 | 0.7744 | 89 | -0.85 | 0.7701 |
| D'Hooghe T, 2019 (12weeks, ASP1707 15mg) | 88 | -0.79 | 0.7658 | 89 | -0.85 | 0.7701 |
| Osuga Y, 2020 (12weeks, Relugolix 10mg) | 101 | -0.50 | 0.7000 | 75 | -0.70 | 0.8000 |
| Osuga Y, 2020 (12weeks, Relugolix 20mg) | 92 | -0.60 | 0.8000 | 75 | -0.70 | 0.8000 |
| Osuga Y, 2020 (12weeks, Relugolix 40mg) | 101 | -0.70 | 0.8000 | 75 | -0.70 | 0.8000 |
| Harada T, 2021 (24weeks, Relugolix 40mg) | 162 | -0.70 | 0.8000 | 156 | -0.80 | 0.8000 |
| Osuga Y, 2021 (24weeks, Relugolix 10mg) | 79 | -0.60 | 0.8100 | 61 | -0.80 | 0.8200 |
| Osuga Y, 2021 (24weeks, Relugolix 20mg) | 74 | -0.70 | 0.8500 | 61 | -0.80 | 0.8200 |
| Osuga Y, 2021 (24weeks, Relugolix 40mg) | 87 | -0.80 | 0.8100 | 61 | -0.80 | 0.8200 |

**Supplementary Table 7.** Extracted data for induration of the Douglas cavity score.

| **Study** | **T sample** | **T mean** | **T sd** | **C sample** | **C mean** | **C sd** |
| --- | --- | --- | --- | --- | --- | --- |
| Acs N, 2015 (12weeks, Elagolix 150mg) | 42 | -2.62 | 2.2035 | 44 | -3.99 | 2.1890 |
| Acs N, 2015 (12weeks, Elagolix 250mg) | 42 | -3.05 | 2.2035 | 44 | -3.99 | 2.1890 |
| Harada T, 2021 (24weeks, Relugolix 40mg) | 171 | -52.60 | 17.0000 | 164 | -57.50 | 17.9290 |

**Supplementary Table 8.** Extracted data for peak score for endometriosis pain.

| **Study** | **T sample** | **T mean** | **T sd** | **C sample** | **C mean** | **C sd** |
| --- | --- | --- | --- | --- | --- | --- |
| Acs N, 2015 (12weeks, Elagolix 150mg) | 42 | -4.40 | 12.9610 | 44 | -10.50 | 13.2660 |
| Acs N, 2015 (12weeks, Elagolix 250mg) | 42 | -8.30 | 12.9610 | 44 | -10.50 | 13.2660 |
| Osuga Y, 2020 (12weeks, Relugolix 10mg) | 103 | -6.60 | 10.8000 | 80 | -8.40 | 12.7000 |
| Osuga Y, 2020 (12weeks, Relugolix 20mg) | 100 | -6.30 | 14.0000 | 80 | -8.40 | 12.7000 |
| Osuga Y, 2020 (12weeks, Relugolix 40mg) | 103 | -10.10 | 13.4000 | 80 | -8.40 | 12.7000 |
| Harada T, 2021 (24weeks, Relugolix 40mg) | 165 | -8.97 | 14.2580 | 156 | -10.80 | 14.1140 |
| Osuga Y, 2021 (24weeks, Relugolix 10mg) | 80 | -6.90 | 10.3900 | 63 | -11.90 | 13.3300 |
| Osuga Y, 2021 (24weeks, Relugolix 20mg) | 77 | -7.80 | 15.1900 | 63 | -11.90 | 13.3300 |
| Osuga Y, 2021 (24weeks, Relugolix 40mg) | 88 | -10.80 | 14.9400 | 63 | -11.90 | 13.3300 |

**Supplementary Table 9.** Extracted data for percentage of days of analgesic use.

| **Study** | **T sample** | **T mean** | **T sd** | **C sample** | **C mean** | **C sd** |
| --- | --- | --- | --- | --- | --- | --- |
| Osuga Y, 2020 (12weeks, Relugolix 10mg) | 103 | -0.60 | 1.2000 | 80 | -2.30 | 0.7000 |
| Osuga Y, 2020 (12weeks, Relugolix 20mg) | 100 | -1.20 | 1.2000 | 80 | -2.30 | 0.7000 |
| Osuga Y, 2020 (12weeks, Relugolix 40mg) | 103 | -2.30 | 0.7000 | 80 | -2.30 | 0.7000 |
| Harada T, 2021 (24weeks, Relugolix 40mg) | 171 | -2.36 | 0.3923 | 164 | -2.50 | 0.3842 |

**Supplementary Table 10.** Extracted data for menstrual blood loss.

| **Study** | **T sample** | **T mean** | **T sd** | **C sample** | **C mean** | **C sd** |
| --- | --- | --- | --- | --- | --- | --- |
| Acs N, 2015 (12weeks, Elagolix 150mg) | 42 | -19.00 | 26.5710 | 44 | -31.80 | 25.8700 |
| Acs N, 2015 (12weeks, Elagolix 250mg) | 43 | -25.00 | 30.8200 | 44 | -31.80 | 25.8700 |
| Osuga Y, 2020 (12weeks, Relugolix 10mg) | 101 | -18.30 | 19.8000 | 75 | -23.30 | 20.5000 |
| Osuga Y, 2020 (12weeks, Relugolix 20mg) | 92 | -17.80 | 20.4000 | 75 | -23.30 | 20.5000 |
| Osuga Y, 2020 (12weeks, Relugolix 40mg) | 101 | -25.30 | 20.9000 | 75 | -23.30 | 20.5000 |
| Harada T, 2021 (24weeks, Relugolix 40mg) | 165 | -28.61 | 17.9830 | 156 | -32.01 | 17.7360 |
| Osuga Y, 2021 (24weeks, Relugolix 10mg) | 79 | -17.00 | 20.2900 | 61 | -26.40 | 20.3400 |
| Osuga Y, 2021 (24weeks, Relugolix 20mg) | 74 | -20.60 | 19.6500 | 61 | -26.40 | 20.3400 |
| Osuga Y, 2021 (24weeks, Relugolix 40mg) | 87 | -25.90 | 19.9000 | 61 | -26.40 | 20.3400 |

**Supplementary Table 11.** Extracted data for Pain.

| **Study** | **T sample** | **T mean** | **T sd** | **C sample** | **C mean** | **C sd** |
| --- | --- | --- | --- | --- | --- | --- |
| Acs N, 2015 (12weeks, Elagolix 150mg) | 42 | -17.90 | 25.2750 | 44 | -23.90 | 32.5030 |
| Acs N, 2015 (12weeks, Elagolix 250mg) | 43 | -20.90 | 33.4430 | 44 | -23.90 | 32.5030 |
| Osuga Y, 2020 (12weeks, Relugolix 10mg) | 101 | -13.70 | 18.7000 | 75 | -19.80 | 23.4000 |
| Osuga Y, 2020 (12weeks, Relugolix 20mg) | 92 | -14.60 | 23.6000 | 75 | -19.80 | 23.4000 |
| Osuga Y, 2020 (12weeks, Relugolix 40mg) | 101 | -17.20 | 22.5000 | 75 | -19.80 | 23.4000 |
| Harada T, 2021 (24weeks, Relugolix 40mg) | 165 | -17.71 | 16.9560 | 156 | -20.42 | 16.7370 |
| Osuga Y, 2021 (24weeks, Relugolix 10mg) | 79 | -14.00 | 17.5000 | 61 | -24.80 | 23.8400 |
| Osuga Y, 2021 (24weeks, Relugolix 20mg) | 74 | -20.00 | 21.8800 | 61 | -24.80 | 23.8400 |
| Osuga Y, 2021 (24weeks, Relugolix 40mg) | 87 | -20.90 | 21.6800 | 61 | -24.80 | 23.8400 |

**Supplementary Table 12.** Extracted data for Control and Powerlessness.

| **Study** | **T sample** | **T mean** | **T sd** | **C sample** | **C mean** | **C sd** |
| --- | --- | --- | --- | --- | --- | --- |
| Acs N, 2015 (12weeks, Elagolix 150mg) | 42 | -10.70 | 26.5710 | 44 | -13.60 | 29.1860 |
| Acs N, 2015 (12weeks, Elagolix 250mg) | 43 | -12.80 | 28.8530 | 44 | -13.60 | 29.1860 |
| Osuga Y, 2020 (12weeks, Relugolix 10mg) | 101 | -8.30 | 16.4000 | 75 | -8.90 | 17.3000 |
| Osuga Y, 2020 (12weeks, Relugolix 20mg) | 92 | -8.90 | 18.6000 | 75 | -8.90 | 17.3000 |
| Osuga Y, 2020 (12weeks, Relugolix 40mg) | 101 | -10.40 | 17.8000 | 75 | -8.90 | 17.3000 |
| Harada T, 2021 (24weeks, Relugolix 40mg) | 165 | -16.39 | 17.5980 | 156 | -15.28 | 17.4860 |
| Osuga Y, 2021 (24weeks, Relugolix 10mg) | 79 | -8.40 | 15.9200 | 61 | -12.40 | 18.3300 |
| Osuga Y, 2021 (24weeks, Relugolix 20mg) | 74 | -15.40 | 17.8600 | 61 | -12.40 | 18.3300 |
| Osuga Y, 2021 (24weeks, Relugolix 40mg) | 87 | -13.30 | 16.3200 | 61 | -12.40 | 18.3300 |

**Supplementary Table 13.** Extracted data for Emotional Wellbeing.

| **Study** | **T sample** | **T mean** | **T sd** | **C sample** | **C mean** | **C sd** |
| --- | --- | --- | --- | --- | --- | --- |
| Acs N, 2015 (12weeks, Elagolix 150mg) | 42 | -16.10 | 28.5150 | 44 | -15.30 | 33.8300 |
| Acs N, 2015 (12weeks, Elagolix 250mg) | 43 | -18.60 | 36.7220 | 44 | -15.30 | 33.8300 |
| Osuga Y, 2020 (12weeks, Relugolix 10mg) | 101 | -6.60 | 10.3000 | 75 | -6.80 | 16.5000 |
| Osuga Y, 2020 (12weeks, Relugolix 20mg) | 92 | -8.40 | 17.0000 | 75 | -6.80 | 16.5000 |
| Osuga Y, 2020 (12weeks, Relugolix 40mg) | 101 | -6.80 | 15.2000 | 75 | -6.80 | 16.5000 |
| Harada T, 2021 (24weeks, Relugolix 40mg) | 165 | -7.38 | 15.2860 | 156 | -7.42 | 15.1130 |
| Osuga Y, 2021 (24weeks, Relugolix 10mg) | 79 | -7.50 | 10.8400 | 61 | -10.50 | 17.9200 |
| Osuga Y, 2021 (24weeks, Relugolix 20mg) | 74 | -13.40 | 17.0600 | 61 | -10.50 | 17.9200 |
| Osuga Y, 2021 (24weeks, Relugolix 40mg) | 87 | -10.30 | 17.1100 | 61 | -10.50 | 17.9200 |

**Supplementary Table 14.** Extracted data for Social Support.

| **Study** | **T sample** | **T mean** | **T sd** | **C sample** | **C mean** | **C sd** |
| --- | --- | --- | --- | --- | --- | --- |
| Acs N, 2015 (12weeks, Elagolix 150mg) | 42 | -7.70 | 29.1630 | 44 | -8.50 | 27.1960 |
| Acs N, 2015 (12weeks, Elagolix 250mg) | 43 | -5.80 | 32.7787 | 44 | -8.50 | 27.1960 |
| Osuga Y, 2020 (12weeks, Relugolix 10mg) | 101 | -5.50 | 11.6000 | 75 | -6.20 | 16.4000 |
| Osuga Y, 2020 (12weeks, Relugolix 20mg) | 92 | -6.30 | 14.9000 | 75 | -6.20 | 16.4000 |
| Osuga Y, 2020 (12weeks, Relugolix 40mg) | 101 | -8.40 | 16.2000 | 75 | -6.20 | 16.4000 |
| Harada T, 2021 (24weeks, Relugolix 40mg) | 165 | -8.51 | 14.9000 | 156 | -7.19 | 14.7380 |
| Osuga Y, 2021 (24weeks, Relugolix 10mg) | 79 | -5.90 | 12.8100 | 61 | -9.40 | 15.5500 |
| Osuga Y, 2021 (24weeks, Relugolix 20mg) | 74 | -10.60 | 15.2600 | 61 | -9.40 | 15.5500 |
| Osuga Y, 2021 (24weeks, Relugolix 40mg) | 87 | -9.70 | 17.7400 | 61 | -9.40 | 15.5500 |

**Supplementary Table 15.** Extracted data for Self‑image.

| **Study** | **Exclusion criteria** |
| --- | --- |
| Acs N, 2015 | Patients were administered a GnRH-a or GnRH-ant, or danazol within 6 months of screening, depot medroxyprogesterone acetate within 3 months of screening or had used hormonal contraception or other hormonal therapy within 1 month of screening, or if they had a history of unresponsiveness to GnRH-a or GnRH-ant treatment. |
| D'Hooghe T, 2019 | Exclusion criteria included treatments that alter gynecological endocrinology, surgery for endometriosis within 4 weeks of study initiation, and the presence of pelvic or gynecological abnormalities. |
| Osuga Y, 2020 | Patients with measurable uterine fibroids (longest diameter ≥3 cm), lower abdominal pain from irritable bowel syndrome or severe interstitial cystitis, thyroid dysfunction, pelvic inflammatory disease, positive Papanicolaou smear, history of hysterectomy or bilateral oophorectomy, or serious cardiovascular, hepatic, renal, or hematologic disorders. |
| Osuga Y, 2021 | Patients were excluded if they experienced treatment- emergent adverse events in the preceding study that made continuation unsafe, were unable to comply with the protocol due to new/ aggravated conditions, showed no efficacy in the preceding study, or developed symptoms of hypoestrogenism. |
| Harada T, 2021 | Patients who use bisphosphonates, GnRH analogs, danazol, aromatase inhibitors, dienogest, sex hormones, anticoagulants, antiplatelets, tranexamic acids, selective estrogen receptor modulators, activated vitamin D, calcitonin, ipriflavone, steroid hormones, vitamin K, teriparatide, or denosumab; previous clinical history of hysterectomy or bilateral oophorectomy; having uterine fibroid requiring treatment; having lower abdominal pain because of irritable bowel syndrome or severe interstitial cystitis; pelvic inflammatory disease; positive cervical cytology; or serious cardiovascular, hepatic, renal, or hematologic disorders. |

**Supplementary Table 16.** Detailed exclusion criteria of included randomized controlled trials.

| **Study** | **Domain** | **Risk judgment** | **Justification** |
| --- | --- | --- | --- |
| Acs N, 2015 | Blinding of outcome assessment | Unclear risk | Although the study was described as double-blind, the publication did not provide sufficient details regarding whether outcome assessors were blinded. |
| Acs N, 2015 | Selective reporting | Unclear risk | The study protocol was registered (ClinicalTrials.gov identifier: NCT00797225); however, insufficient information was available to determine whether all prespecified outcomes were reported. |
| Acs N, 2015 | Other bias | Unclear risk | The study was supported by pharmaceutical companies, and potential influence related to sponsor involvement could not be completely excluded. |
| D'Hooghe T, 2019 | Other bias | Unclear risk | The study was funded by Astellas Pharma Inc., and several authors had affiliations or financial relationships with the sponsor; therefore, potential influence related to funding could not be completely excluded. |
| Harada T, 2021 | Blinding of outcome assessment | Unclear risk | Although the study reported a double-blind design, details regarding blinding of outcome assessors were insufficiently described; therefore, the risk of detection bias was considered unclear. |
| Harada T, 2021 | Other bias | Unclear risk | The study was supported by ASKA Pharmaceutical Co., Ltd., and some investigators reported employment or consulting relationships with the sponsor. The potential impact of sponsor involvement could not be excluded. |
| Osuga Y, 2020 | Other bias | Unclear risk | The study was supported by Takeda Pharmaceutical Company Limited, which was involved in study design, protocol development, data collection, review, and analysis. The potential influence of sponsor involvement was therefore considered unclear. |
| Osuga Y, 2021 | Selective reporting | Unclear risk | This was an extension study of a previous randomized trial. Although outcomes were reported, insufficient information was available to determine whether all prespecified outcomes were fully reported. |
| Osuga Y, 2021 | Other bias | Unclear risk | The study used an open-label extension design, and the potential influence of the non-blinded design on outcome assessment could not be excluded. |

**Supplementary Table 17.** Detailed justification for unclear risk-of-bias judgments.

# 1 Reference

1. Kim S, Chen J, Cheng T, Gindulyte A, He J, He S, et al. PubChem 2023 update. Nucleic Acids Res (2023) 51: D1373-d80. doi: 10.1093/nar/gkac956
